# Mimetic Core‐Sheath Silk Sutures with Superior‐Strength for Pain Management, Anti‐Infection and Wound Healing

**DOI:** 10.1002/adhm.202600024

**Published:** 2026-07-11

**Authors:** Yi Peng, Kai Ma, Xiaoxiao Li, Xiaozhuo Wu, Yuezhou Zhang, Ying Luo, Chenxing Zhang, Wen Zhong, Chunjing Yu, Lin Lu, Malcolm Xing, Guozhong Lyu

**Affiliations:** ^1^ Wuxi School of Medicine Jiangnan University Wuxi China; ^2^ Engineering Research Center of the Ministry of Education for Wound Repair Technology.Jiangnan University The Affiliated Hospital of Jiangnan University Wuxi China; ^3^ Department of Mechanical Engineering University of Manitoba Winnipeg Canada; ^4^ Department of Biosystems Engineering University of Manitoba Winnipeg Canada; ^5^ Department of Nuclear Medicine The Affiliated Hospital of Jiangnan University Wuxi China; ^6^ Department of Plastic and Reconstructive Surgery Shanghai Ninth People's Hospital Shanghai Jiao Tong University School of Medicine Shanghai China

**Keywords:** anti‐bacterial and pro‐healing suture, mimetic core‐shell silk hydrogel sutures, pain management, PET/CT of pain‐related regions, superior strength and steel‐like modulus

## Abstract

Sutures are crucial for tissue approximation and wound healing. Besides their strength limits requirement, they often fail to effectively address complications like infection and pain. Inspired by natural silk's “core‐sheath” structure, our suture features a wet‐spun core of n zinc‐fortified silk fibroin fibers (RSF‐Zn^2+^) after drawing with over 800 MPa strength and around steel‐like 30GPa modulus, forming a robust structure (TZS_n_). The outer shell is a photo‐crosslinked methacrylated sericin (SerMA) with Prilocaine‐ (PSTZS_12_) for pain relief. PSTZS_12_ (215 micrometers) can endure a maximum tensile force of 13.78 N which is double that of commercial 5‐0 Mersilk sutures (235 micrometers). Our silk mimetic hydrogel suture PSTZS_12_ reaches superior strength coordinating with pain‐control (prilocaine), antimicrobial (Zn^2+^), and wound repair ability (silk). PET/CT studies of a rat pain model show that the sutures reduce the metabolic levels of ^18^F‐FDG in 14 pain‐related brain regions, including the cingulate cortex and somatosensory cortex, directly impacting pain management. The suture promotes fibroblast migration, angiogenesis and accelerates wound healing.

## 1 Introduction

Surgical sutures are effective in closing wounds, preventing bacterial infection deep in the tissue and promoting wound healing. However, inflammation post‐surgery, along with foreign body reactions and hyperfibrosis following suture placement, can complicate the healing process [1]. Postoperative pain is another concern, typically intensifying at the incision site on the first day after surgery and potentially leading to severe patient discomfort. Persistent pain can disrupt vital signs, resulting in shortness of breath, elevated blood pressure, insomnia, and a weakened immune response [2, 3]. Clinically, analgesics are frequently employed for postoperative pain relief; however, their systemic use can engender resistance and adverse effects such as lethargy, dizziness, gastric ulcers, renal impairment, and leukopenia. Wound infection, a prevalent challenge following surgical interventions, often stems from bacterial colonization that can lead to localized or widespread infection symptoms [4]. Although antibiotics have been crucial in mitigating the risk of infection, their impact has been diminishing due to the rise of multi‐drug resistant bacteria [5].

Technological progress has broadened the range of suture materials available. Traditional silk sutures, though widely used, present high friction, low strength, significantly inflammatory effect [6], liability to infections and delayed healing [7]. Synthetic polymer sutures, such as Polypropylene, are typically monofilament and non‐absorbable, yet their application has low knot strength, and challenges in handling [6]. In recent years, multifunctional sutures with advanced features—such as self‐electrogenesis [8] and ROS responsiveness [9]—have been introduced. However, the interplay of infection, inflammation, and pain exacerbates the wound's pathological state, slowing down the healing process [10, 11]. Consequently, there is a growing demand for innovative surgical sutures that are designed with advanced functions. To achieve this, a variety of bioactive components and therapeutic agents are being integrated into suture design through techniques like surface coatings and targeted loading, marking a significant advancement in the field of wound care and surgical therapy [12, 13].

Silk, composed of fibroin (60%–80%) and sericin (15%–35%) [14], combines mechanical resilience from fibroin's semi‐crystalline structure and fiber protection from sericin [15]. This blend provides silk with excellent mechanical properties, controlled biodegradability, and exceptional biocompatibility, making it indispensable in biomedicine [16]. Significant research has been conducted to isolate and dissolve fibroin proteins [17], such as using concentrated LiBr solutions to disrupt the hydrogen‐bond network within native silk fibers [18]. This processed silk fibroin can be dissolved and reconstituted into various forms, such as gels, films, sponges, fibers, and other related forms. Formic acid dissolves silk fibroin and shields electrostatic repulsion in concentrated solutions, promoting silk fibroin self‐assembly [19]. However, methanol is recognized as the coagulation for the recrystallization of silk fibroin for water‐insoluble membrane, as it rapidly promotes the formation of hydrophobic crystalline domains of β‐sheet in regenerated silk fibroin [20]. Various regenerated materials such as hydrogels, fibers, microspheres, films, and nanoparticles can be prepared by the characteristics.

As a classic biomaterial design paradigm, the core–shell architecture offers four pivotal advantages: (i) interfacial functionalization to enhance biocompatibility and cell adhesion [21]; (ii) compartmentalized multifunctionality enabling spatiotemporal integration of mechanical support, antibacterial activity, and pro‐healing capacity [22]; (iii) controlled release kinetics via shell‐mediated barrier effects [23]; and (iv) synergistic mechanical reinforcement through rational core–shell material selection [24]. Given the complexity of clinical wound healing involving multicellular and multifactorial dynamics, harnessing these attributes allows for rational optimization of material performance and biological functionality, offering a robust strategy for advanced wound repair biomaterials.

Our biomimetic suture addresses the issue of bacterial colonization in conventional braided silk sutures by integrating antimicrobial zinc ions in core silk‐fibroin fibers and analgesic Prilocaine into SerMA coating for pain management and offers superior mechanical performance. Zinc ions are essential for human physiology, possessing antimicrobial properties and modulating inflammation by inhibiting the NF‐κB signaling pathway [25, 26, 27]. They also promote wound healing by stimulating vascular endothelial growth factors and regulating extracellular protein expression [28, 29]. In our suture design, zinc ions strengthen hydrogel fibers by forming ionic interactions with serine residues in silk fibroin [30, 31]. Prilocaine, frequently employed for local analgesic anesthesia, offers effective pain relief, contributing to improved patient comfort during and post‐surgery [32].

**FIGURE 1:**
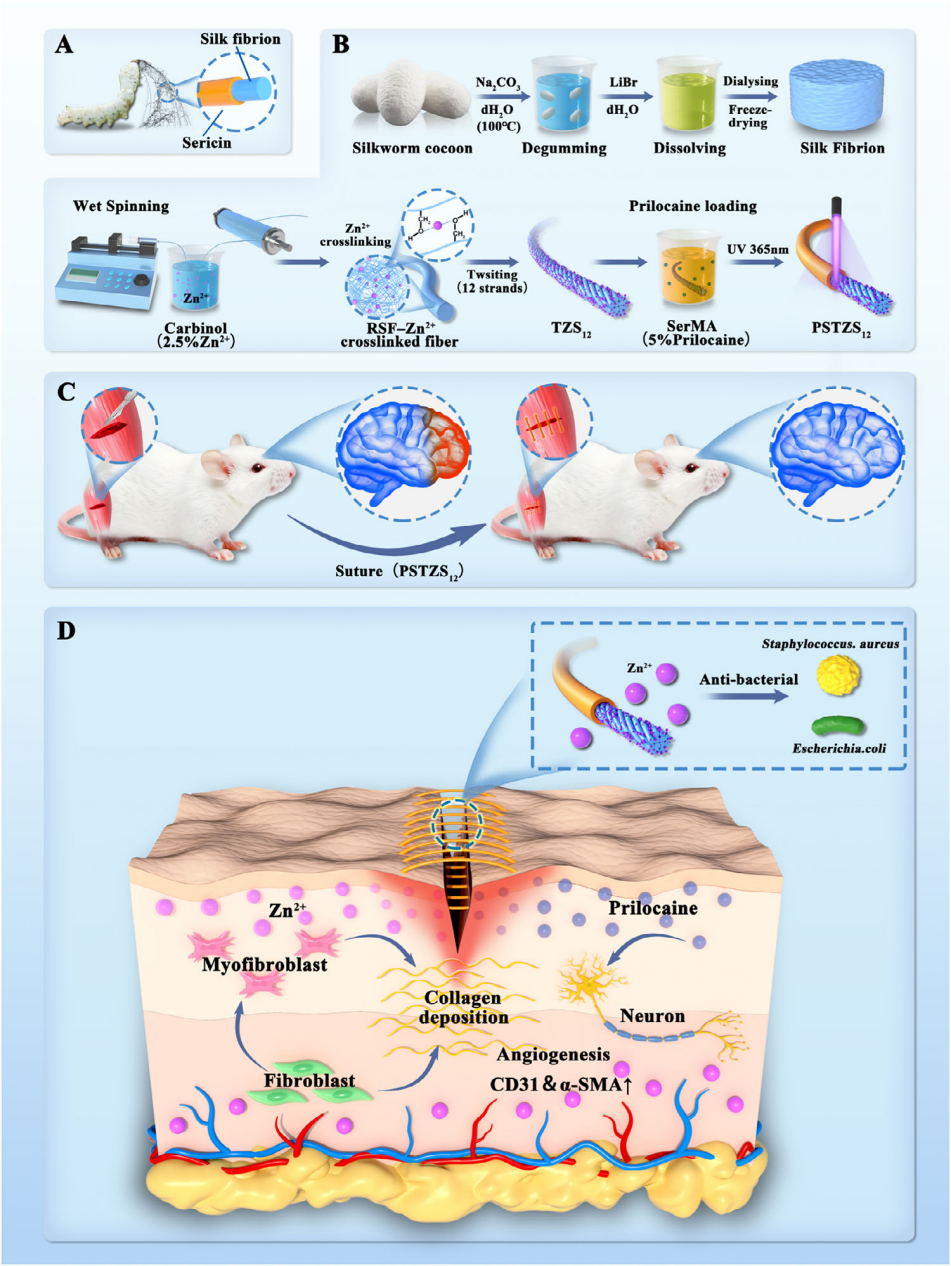
Manufacturing process of PSTZS_12_ multifunctional suture (pain relief, antimicrobial and wound‐healing) (A). The core‐shell structure of natural silk (with sericin as the outer layer and silk fibroin as the inner layer). (B). Fabrication process of PSTZS_12_. (C). Rat quadriceps cut and the analgesic effect evaluation. D. Skin healing, antibacterial activity, and analgesia of PSTZS_12_.

As shown in Figure 1, we developed silk‐mimetic multifunctional hydrogel suture. The hydrogel fiber of the suture is prepared by process of wet‐spinning followed by deep drawing and Zn^2+^ coordination. This advanced suture is encased in a sheath of methacrylic anhydrided sericin (SerMA) and incorporates the analgesic Prilocaine for enhanced pain management. The suture's core, known as TZS_12_, is meticulously crafted by twisting twelve strands of regenerated silk protein hydrogel fibers. The integration of zinc ions and the Prilocaine within a durable SerMA coating positions our suture as a cutting‐edge solution in the realm of integrated wound management, promising superior performance and enhanced patient safety. In addition, our objective assessment of pain in rat brain regions by animal behavior and PET/CT [33]. The assessment of the wound‐healing‐promoting effects of PSTZS_12_ was carried out using an incision model.

## 2 Results

### 2.1 Characterization of Fibers in General

**FIGURE 2:**
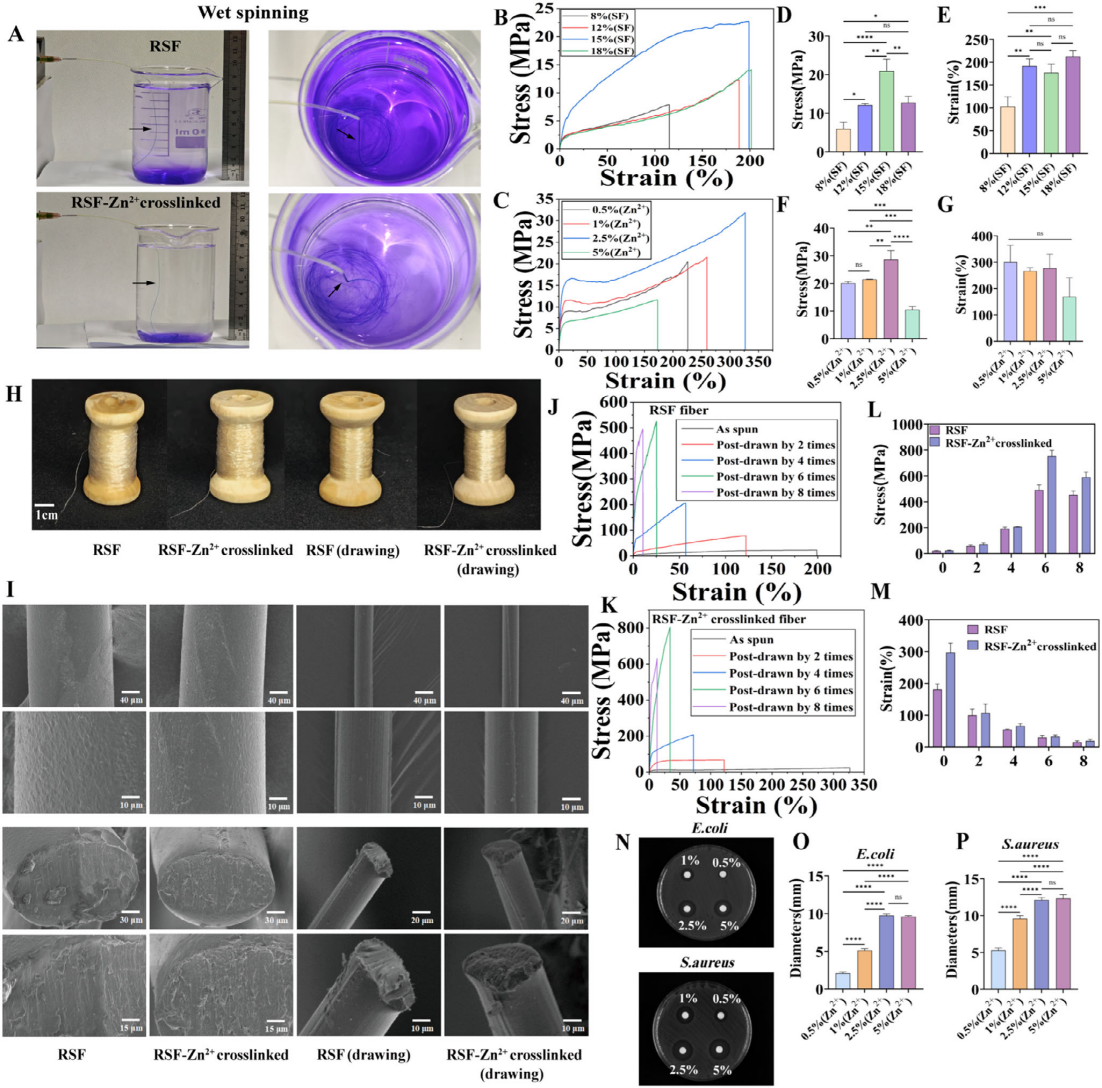
Wet‐spinning preparation of regenerated silk fibroin fibers and their mechanical, antimicrobial, and electron microscopic characterizations. (A). Wet‐spinning preparation of RSF/RSF‐Zn^2+^ cross‐linked fibers. (B). Stress‐strain curves for RSF hydrogel fibers with varying silk fibroin protein concentrations. (C). Stress‐strain curves for RSF fibers cross‐linked with different zinc ion concentrations. (D, E). Statistical analysis of stress and strain in RSF fibers with various concentrations. (F, G). Statistical analysis of stress and strain in RSF fibers cross‐linked with different zinc ion concentrations. (H). Photographs of RSF, RSF‐Zn^2+^cross‐linked, RSF(drawing), and RSF‐Zn^2+^ crosslinked (drawing) fibers. (I). SEM characterization of the surface and cross‐section of RSF, RSF‐Zn^2+^crosslinked RSF(drawing) and RSF‐Zn^2+^crosslinked (drawing) fibers. (J, K). Stress and strain curves for RSF and RSF‐Zn^2+^crosslinked fibers with different drawing ratios. (L, M). Statistical analysis of stress and strain for RSF and RSF‐Zn^2+^crosslinked fibers with various drawing multiples. (N). Inhibition of Staphylococcus aureus and Escherichia coli by zinc ion loading at different concentrations. (O, P). Statistical analysis of the inhibition zones of Staphylococcus aureus and Escherichia coli at various zinc ion concentrations. All experiments were performed in triplicate (N = 3). Data are expressed as mean±standard deviation (SD).

As shown in Figure 2A, Video S1A,B, wet‐spinning was used to prepare RSF fibers and RSF‐Zn^2+^crosslinked fibers via a 200 µm‐inner‐diameter tube. To facilitate the observation of the wet‐spinning process, we added the dye (Methyl Violet) to the spinning solution. The fibers were solidified in the coagulation bath for 10 min before being used for testing. As illustrated in Figure 2B–G), we conducted a comprehensive evaluation of how different concentrations of spinning solutions influence the properties of the fibers. The maximum stresses of fibers (diameter = 199.33 ± 3.05 µm) prepared from RSF spinning solutions at concentrations of 8%, 12%, 15%, and 18% were 7.89 MPa, 12.32 MPa, 22.73 MPa, and 14.14 MPa, respectively. Compared with the 8% group, the 12%, 15%, and 18% groups showed no significant difference in strain. Our findings revealed that hydrogel fibers produced from a 15% silk fibroin (SF) solution exhibited the most superior mechanical performance. Conversely, a higher concentration of 18% SF led to non‐uniform fibers due to high viscosity, which negatively impacted their mechanical attributes. Moreover, we delved into the impact of varying zinc ion crosslinking concentrations on the fibers' mechanical properties. The fibers (diameter = 197.66 ± 2.51 µm) crosslinked in methanol coagulation baths with Zn^2+^ concentrations of 0.5%, 1%, 2.5%, and 5% exhibited maximum stresses of 20.54, 21.52, 31.91, and 11.65 MPa, respectively. The 0.5%, 1%, 2.5%, and 5%groups showed no significant difference in strain. We observed that the optimal stress‐strain behavior was achieved at a 2.5% zinc ion concentration. However, increasing the concentration to 5% led to greater fiber stiffness, likely attributable to the excessive presence of zinc ions.

RSF‐Zn^2+^ crosslinked fibers need to be prepared in a coagulation bath and recrystallization bath containing zinc ions, and the other steps are the same as above. As shown in Figure 2H, the regenerated silk fibroin fibers appear transparent under visible light. After being drawing, they become finer and acquire a certain metallic luster. Scanning electron microscopy (SEM), as shown in Figure 2I, reveals the morphological effects of drawing and zinc ion cross‐linking on the fibers. Fibers without drawing have uniform diameters of approximately 200 µm, characterized by fish scale‐like surface pattern. However, after undergoing 6‐fold drawing process, the fibers' diameter refines to about 40 µm, and the surface evolves into an oriented stripe pattern, indicative of the internal crystallization's orientation.

We further examined the stress‐strain behavior of fibers in a methanol solidification bath (Figure 2J) and fibers in a 2.5% ZnCl_2_ methanol solidification bath (Figure 2K) with varying levels of drawing. Figure 2L illustrates that as drawing increases, so does the fiber stress. The maximum stress for the 6‐fold drawing RSF fiber and RSF‐Zn^2+^ crosslinked fiber reached 525 MPa and 804 MPa, respectively. However, an increase in drawing to 8 times resulted in a decrease in stress, potentially due to excessive physical drawing leading to changes in the internal spatial structure, causing strain or even fracturing of some chemical bonds. Figure 2M demonstrates that hydrogel fibers without drawing possess excellent plasticity, with maximum strains for RSF fibers and RSF‐Zn^2+^ crosslinked fibers being 198.94% and 284.22%, respectively. Nevertheless, as the number of drawing applications increases, the strain decreases, likely due to the increased orientation of internal crystallization and a corresponding decrease in plasticity. Thus, zinc ion crosslinking and drawing ratio can enhance the mechanical properties of the filamentous fibers, which the fiber with 6‐fold drawing is the maximum of stress. Table 1 shows the elastic modulus of fibers at various drawing ratios. The modulus of RSF fiber rises with increasing drawing ratio, exceeding 18 GPa at 8‐fold drawing. RSF‐Zn^2+^crosslinked fiber reaches 40 GPa at 6‐fold drawing, but its modulus decreases at 8‐fold drawing.

**TABLE 1:** Elastic Modulus of Fibers at Different Drawing Ratios (GPa).

Drawing Ratio	0	2	4	6	8
RSF fiber RSF‐Zn^2+^crosslinked fiber	0.080 ± 0.008 0.088 ± 0.025	0.828 ± 0.093 1.070 ± 0.331	3.118 ± 0.331 3.235 ± 0.203	16.687 ± 4.071 37.345 ± 6.599	18.345 ± 1.676 26.466 ± 2.320

Data are expressed as mean±standard deviation (SD), N = 3.

In Figure 2N–P, we evaluated the maximum zinc ion adsorption capacity of RSF‐Zn^2+^ crosslinked fibers in different Zn^2+^ solution concentrations and validated the results using inhibition zone assays. We found that the inhibitory zones against Staphylococcus aureus and Escherichia coli expanded with increasing zinc ion concentrations. However, the largest inhibitory zones were observed at a 2.5% zinc ion concentration. The inhibitory zones at 5% were similar to those at 2.5%, suggesting that the adsorption capacity of RSF‐Zn^2+^ fibers for zinc ions plateaus at a 2.5% concentration.

**FIGURE 3:**
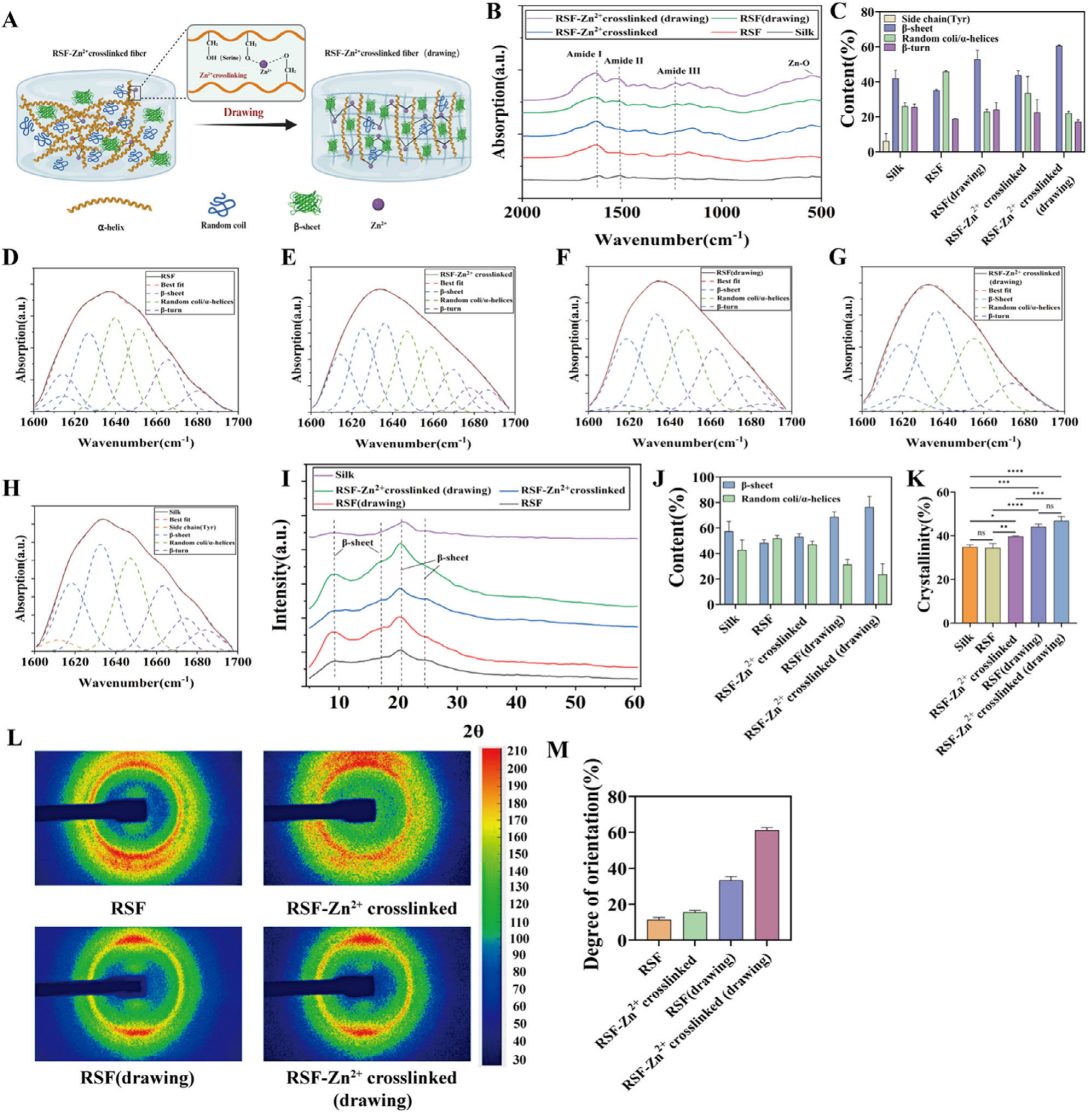
Secondary structure analysis of the fibers. (A). The secondary structure changes of RSF‐Zn^2+^crosslinked fiber before and after drawing (Created in BioRender. Peng, Y. (2026) https://BioRender.com/fp5vf28). (B). FTIR of silk, RSF, RSF‐Zn^2+^crosslinked, RSF(drawing), RSF‐Zn^2+^crosslinked(drawing). (C). Statistics of the component content in the amide I of silk, RSF, RSF(drawing), RSF‐Zn^2+^crosslinked, RSF‐Zn^2+^crosslinked(drawing). (D–H). Amide I peak fitting curve of RSF (D), RSF‐Zn^2+^crosslinked (E), RSF(drawing) (F), RSF‐Zn^2+^crosslinked(drawing) (G), Silk(H). I. XRD of silk, RSF, RSF‐Zn^2+^crosslinked, RSF(drawing), RSF‐Zn^2+^crosslinked(drawing). J. Statistics of the β‐sheet and random coli/α‐helix of silk, RSF/RSF(drawing), RSF‐Zn^2+^crosslinked, RSF‐Zn^2+^crosslinked(drawing) in the amide II. K. Crystallinity statistics of silk, RSF/RSF(drawing), RSF‐Zn^2+^crosslinked, RSF‐Zn^2+^crosslinked(drawing). (L). 2D‐WAXS patterns of RSF/RSF(drawing), RSF‐Zn^2+^crosslinked, RSF‐Zn^2+^crosslinked(drawing). (M). Orientation degree analysis of RSF/RSF(drawing), RSF‐Zn^2+^crosslinked, RSF‐Zn^2+^crosslinked(drawing). All experiments were performed in triplicate (N = 3). Data are expressed as mean±standard deviation (SD).

As illustrated in Figure 3A, the drawing aligns the protein chains and significantly increases the number of hydrogen bonds between them. Zinc ions coordinate and crosslink with serine to form an ionic network, thereby consolidating the mechanical properties [30]. We recorded and analyzed the unique spectral signatures of RSF fiber, RSF (drawing) fiber, RSF‐Zn^2+^crosslinked fiber, RSF‐Zn^2+^crosslinked (drawing) fiber, as well as degummed silk fiber using a Fourier‐transform infrared (FTIR) spectrometer. In Figure 3B, the amide I band [34], a hallmark of β‐sheet structure, was prominently displayed at 1630 cm^−1^, while the amide II band [34], indicative of β‐crystallization, appeared at 1510 cm^−1^. Additionally, the amide III band [34], also reflective of β‐sheet structure, was observed at 1230 cm^−1^, and the absorption peak corresponding to the coordination structure formed by Zn‐O bonds [30] was noted at 542 cm^−1^. The intensity of the amide II band was significantly amplified by the drawing process, suggesting that under gentle stretching, SF underwent a conformational transition from a relatively unordered structure to one with abundant β‐sheet antiparallel configurations [35].

Further, we performed a Fourier deconvolution and second‐order derivative fitting on the amide I band to dissect the peaks [36], as detailed in Figure 3D–H. This analysis provided a deeper understanding of the secondary structures present in degummed natural silk, RSF, RSF(drawing), RSF‐Zn^2+^crosslinke and RSF‐Zn^2+^crosslinked(drawing). As in Figure 3C, the degummed silk fiber exhibited a β‐sheet content of approximately 42.04%. In comparison the β‐sheet contents for RSF, RSF(drawing), RSF‐Zn^2+^crosslinked, and RSF‐Zn^2+^crosslinked (drawing) fibers were approximately 35.13%, 52.84%, 43.75%, and 60.68%, respectively. These results underscore that the formation of β‐sheets is significantly enhanced by both zinc ion cross‐linking [30] and the drawing process, aligning with the observed improvements in the fibers' mechanical properties [37].

Furthermore, X‐ray diffraction (XRD) analysis, as presented in Figure 3I, revealed the characteristic β‐sheet peaks of amide II for RSF, RSF(drawing), RSF‐Zn^2+^crosslinked, RSF‐Zn^2+^crosslinked(drawing), and degummed natural silk at angles of 9.1°, 18.9°, 20.7° and 24 [38]. The prominence of the β‐sheet characteristic peaks was notably intensified by the drawing process, indicating a structural transformation toward a more ordered β‐sheet configuration.

Through the application of second‐order derivative split‐peak fitting statistics on the XRD curves, depicted in Figure 3J, we determined that the amide II β‐sheet (crystalline) content of degummed silk fiber was approximately 57.30%. Comparatively, the amide II β‐folding (crystalline) content for RSF, RSF(drawing), RSF‐Zn^2+^crosslinked, RSF‐Zn^2+^crosslinked(drawing) was found to be approximately 48.26%, 68.69%, 52.92%, and 76.42%, respectively. These results suggest that zinc ion cross‐linking can moderately enhance the total β‐folding content, while the drawing robustly promotes the generation of total β‐folding and diminishes the presence of random coils and α‐helices. This shift in molecular structure enhances the fibers' rigidity and toughness, consequently improving their mechanical properties.

Figure 3K demonstrates that the crystallinity of RSF is essentially equivalent to that of degummed natural silk. However, the crystallinity of RSF that has undergone zinc ion cross‐linking and drawing is markedly higher than that of the untreated RSF and degummed natural silk. This observation underscores that zinc ion cross‐linking and the drawing process can significantly amplify the crystallinity of the fibers, thereby enhancing their mechanical integrity and performance. Figure 3L presents 2D‐WAXS patterns of the four fiber variants. RSF and RSF‐Zn^2+^cross‐linked fibers exhibit concentric Debye rings, indicative of an isotropic, randomly oriented supramolecular architecture. Upon drawing, these rings collapse into well‐defined equatorial arcs, evidencing pronounced molecular alignment along the fiber axis. The orientation factor of RSF and RSF‐Zn^2+^cross‐linked fibers was 12.86% and 16.52%, respectively, whereas that of the drawn RSF/RSF‐Zn^2+^cross‐linked fibers reached 35.68% and 62.56% (Figure 3M). Quantitative orientation analysis reveals that the Herman orientation parameter of the drawn RSF/RSF‐Zn^2+^cross‐linked fibers markedly surpasses that of their undrawn counterparts; Zn^2+^‐mediated cross‐linking further amplifies this orientational enhancement. Collectively, the data demonstrate that mechanical drawing orchestrates a transformation from a disordered, isotropic hydrogel network to a highly oriented, anisotropic crystalline superstructure, thereby substantially upgrading the mechanical performance of the fibers.

### 2.2 Characterization of Suture Fabrication

We finally decided to utilize wet spinning with a 2.5% Zn^2+^ methanol coagulation bath to create fibers with drawing ratio and infused with zinc ions, serving as the core for our sutures. This process enhances the mechanical properties of the fibers, making them suitable for suture applications. In addition, the methacrylic anhydride‐coated sericin protein (SerMA) is recognized for its excellent biocompatibility and can be cross‐linked through UV polymerization, which eliminates the risks associated with chemical cross‐linking while providing adhesion sites for cell attachment [39]. The SerMA as the suture sheath has a two‐fold function: (1) to mimic raw silk using sericin to protect silk fibroin fibers and glue the core together and (2) the sheath's porous structure creates an ideal environment for the controlled release of therapeutic drugs.

Furthermore, SerMA fills the gaps between the fibers, increasing their overall strength, and smooths the suture surface to reduce resistance during wound closure. In the final assembly, the SerMA solution, combined with Prilocaine, was applied to the sutures and exposed to UV light for cross‐linking. This innovative approach ensures that the sutures not only provide essential mechanical support but also deliver therapeutic agents, offering a dual benefit in biomedical applications.

**FIGURE 4:**
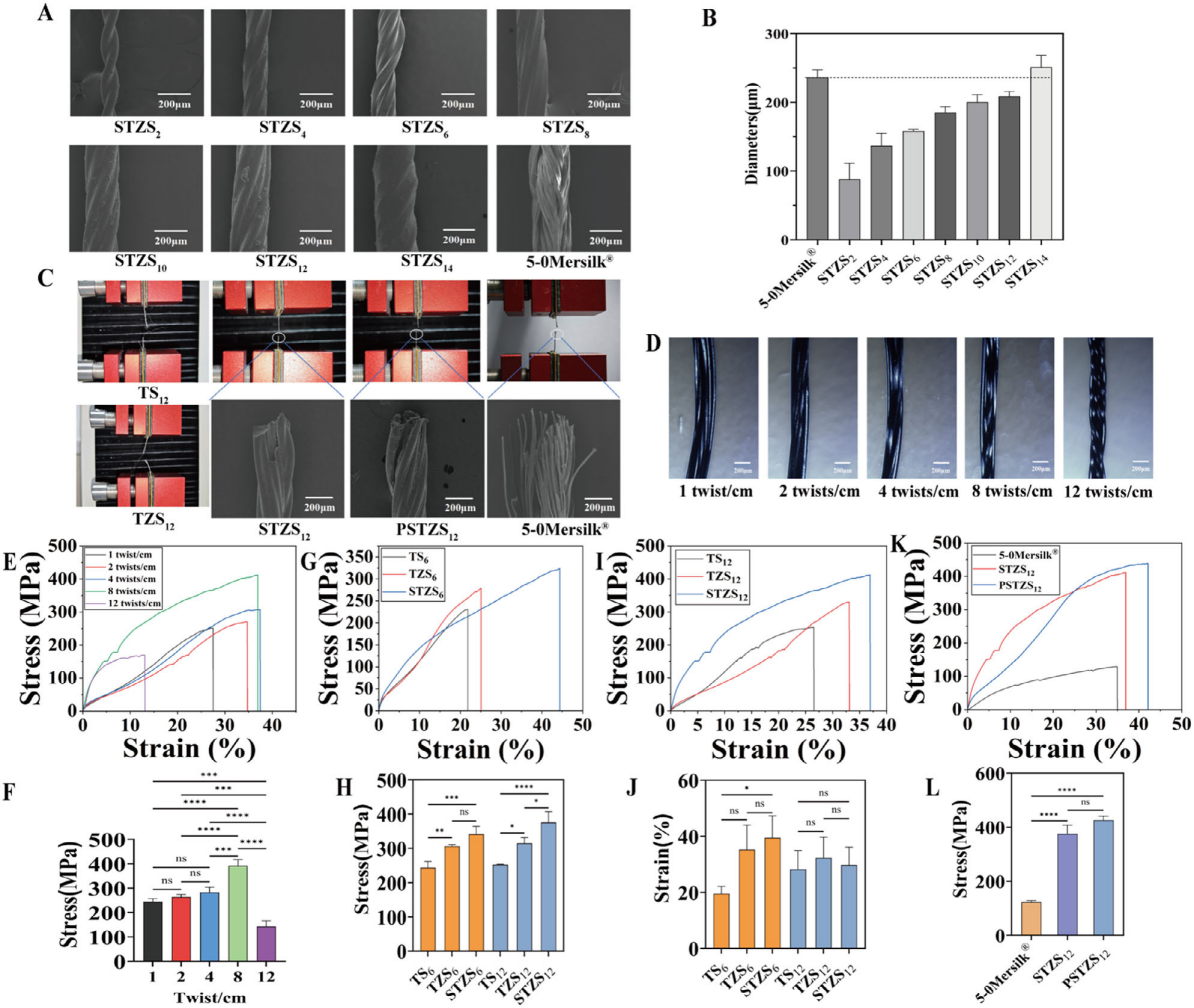
Mechanical testing and performance of the sutures. (A). Electron microscopy characterization of STZS sutures with customized strand counts and the comparison with the 5‐0 Mersilk suture. (B). Statistics of diameters for the 5‐0 Mersilk suture and customized strands of STZS. (C). Fracture cross‐sections after tensile tests of TS_12_, TZS_12_, STZS_12_, PSTZS_12_, and 5‐0 Mersilk sutures. (D). Photomicrographs of sutures (12 strands of fibers) with varying degrees of twist. (E, F). Twist optimization and mechanical comparison: Evaluation of the stress‐strain relationship and mechanical properties in STZS_12_ sutures with different twists. (G, H, I, J).Stress‐strain relationships and mechanical properties of TS, TZS, and STZS sutures with 12 and 6 strands during tensile tests. All sutures were twisted at 8 twists per centimeter(T/cm) except for the 5‐0 Mersilk suture. (K, L). Stress‐strain curves and stress levels of STZS_12_, PSTZS_12_ and 5‐0Mersilk sutures. All experiments were performed in triplicate (N = 3). Data are expressed as mean±standard deviation (SD).

We conducted experiments with varying numbers of strands of STZS, as shown in Figure 4A,B, and found that a bundle of 12 strands of STZS had a diameter of approximately 215 µm. We selected 5‐0 Mersilk as a control due to its status as a natural, non‐absorbable braided silk suture. This suture is known for its excellent biocompatibility, achieved through a degumming process that removes proteins responsible for foreign body reactions. Its surface is coated with wax to enhance strength, improve flexibility, and reduce the coefficient of friction. Therefore, it is widely used in clinical practice. Nevertheless, braided sutures made from silk materials may pose a risk of introducing pathogens into the wound, potentially leading to infection [14]. It is also worth noting that Mersilk sutures are monofunctional, lack antimicrobial and analgesic properties.

Figure 4C and Video S3 illustrate the fracture interfaces of TS_12_, TZS_12_, and 5–0 Mersilk sutures. The twisting process leaves gaps, and during mechanical testing, these sutures tend to break one fiber by one fiber at a time. In contrast, the SerMA coating fills the spaces between the fibers in STZS_12_ and PSTZS_12_, leading to a uniform fracture pattern. The internal fibers are tightly bonded, allowing the fracture force to be evenly distributed among them, resulting in a clean and uniform fracture interface with high fracture strength [40].

The 5–0 Mersilk sutures, being braided, enable the internal fibers to share the fracture force, producing a rough fracture area. These braided sutures exhibit a loose internal structure relative to their outer diameter, which may affect their overall performance and integrity.

The degree of twist in a suture significantly influences its mechanical properties, a factor we explored by preparing STZS_12_ sutures with twists ranging from 1 to 12 per centimeter (T/cm) for our tests. Microscopic observations of the STZS_12_ twists are presented in Figure 4D. We examined the effects of varying twist levels on STZS_12_. Figure 4E,F illustrate that the maximum stress values at twist levels of 1, 2, 4, 8, and 12 twists/cm were 251.94 MPa, 270.63 MPa, 307.08 MPa, 412.15 MPa, and 169.60 MPa, respectively. These findings indicate that the maximum stress increases with twist level up to 8 twists/cm, where it reaches a peak. However, due to excessive twisting causing fiber wear, STZS_12_ exhibited a notable stress decline at 12 twists/cm.

In an extended comparison of the mechanical properties of TS_12_, TZS_12_, STZS_12_, TS_6_, TZS_6_ and STZS_6_ at the 8 T/cm twist level, both STZS_12_ and STZS_6_ demonstrated high mechanical strengths following SerMA UV curing. For a more targeted comparison, we selected STZS_12_ and STZS_6_ to validate stress‐strain behavior. The stress‐strain curves shown in Figure 4G,I indicate that the maximum stresses of TS_12_, TZS_12_, and STZS_12_ were 253.50 MPa, 330.47 MPa, and 412.15 MPa, respectively. Meanwhile, the maximum stresses of TS_6_, TZS_6_, and STZS_6_ were 257.93 MPa, 311.47 MPa, and 361.43 MPa, respectively. As shown in Figure 4H, STZS_12_ can endure maximum stresses 1.25 to 1.5 times higher than those of TS_12_ and TZS_12_. Meanwhile, the maximum stresses that STZS_6_ can withstand are 1.25 to 1.5 times greater than those of TS_6_ and TZS_6_.

However, regarding the strains shown in Figure 4J, no significant difference is observed between the 12‐strand and 6‐strand groups, indicating that other factors, such as twist level and cross‐linking, are more critical in determining the mechanical performance of the sutures.

Armed with the knowledge of the optimal twist at 8 T/cm, Figure 4K,L display the stress‐strain curves and stress intensity for STZS_12_, PSTZS_12_, and 5‐0 Mersilk sutures. Among them, The tensile strength of STZS_12_ and PSTZS_12_ can achieve 12.94 N (equivalent to around 412 MPa) and 13.78 N (equivalent to around 438 MPa), respectively. In comparison, 5–0 Mersilk exhibits much weaker mechanical performance, with a tensile strength of only 5.93 N (equivalent to approximately 126 MPa). Mechanical performance evaluation under high‐humidity conditions demonstrated that water‐swollen STZS_12_ and PSTZS_12_ sutures retained tensile strengths of approximately 250 MPa (Figure S1A), representing a 2.5‐fold enhancement over 5‐0 Mersilk (approximately 100 MPa). Notably, this performance was achieved with only modest reduction from dry‐state values and without significant alteration to deformation behavior (Figure S1B,C). Our analysis revealed that while the fracture forces of STZS_12_ and PSTZS_12_ are similar, they are significantly more robust than that of the 5‐0 Mersilk suture.

**FIGURE 5:**
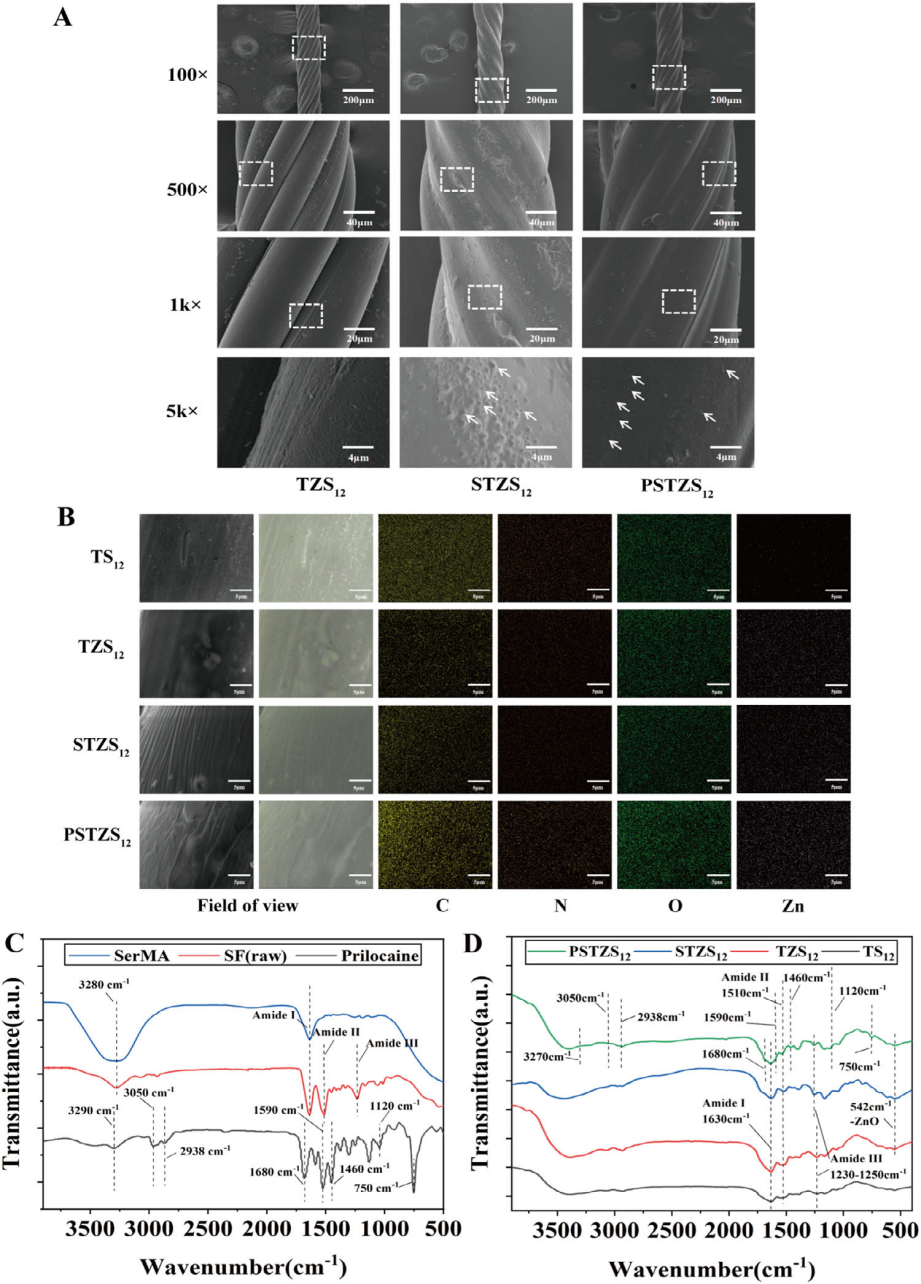
Electron microscopy characterization, EDS and FTIR of the sutures and materials. (A). Scanning electron microscopy characterization at different magnifications of TZS_12_, STZS_12_ and PSTZS_12_. (B). EDS tests of TS_12_, TZS_12_, STZS_12_, PSTZS_12_. (C).FTIR test of SF(raw), Sericin‐MA, Prilocain. D. FTIR test of TS_12_, TZS_12_, STZS_12_, PSTZS_12_.

SEM depicted in Figure 5A elucidates the morphological transformations from TZS_12_ to STZS_12_ and then to PSTZS_12_. The inner fibers of TZS_12_ are smooth. In contrast, STZS_12_ and PSTZS_12_ are coated with SerMA, and the pores of the coating (indicated by white arrows) are designed to facilitate the release of the drug.

Energy‐dispersive X‐ray spectroscopy (EDS) was employed to analyze the elemental composition of PSTZS_12_, STZS_12_, TZS_12_, and TS_12_, as shown in Figure 5B. The significantly higher content of zinc elements in PSTZS_12_, STZS_12_, and TZS_12_ relative to TS_12_ confirms the successful adsorption of zinc ions onto the fiber, revealing the antibacterial potential of the sutures.

Figure 5C,D show the FTIR analysis of SerMA, raw RSF, Prilocaine and different sutures. (For details, S1.) These findings demonstrate that zinc ions can form ionic covalent bonds with the serine of the silk fibroin protein, and Prilocaine can be effectively loaded within the SerMA coatings and crosslinked to the sutures, offering a robust platform for the integration of therapeutic agents into the suture material and enhancing their therapeutic potential.

### 2.3 Drug Release and Cytotoxicity and In Vivo Bio‐Compatibility

**FIGURE 6:**
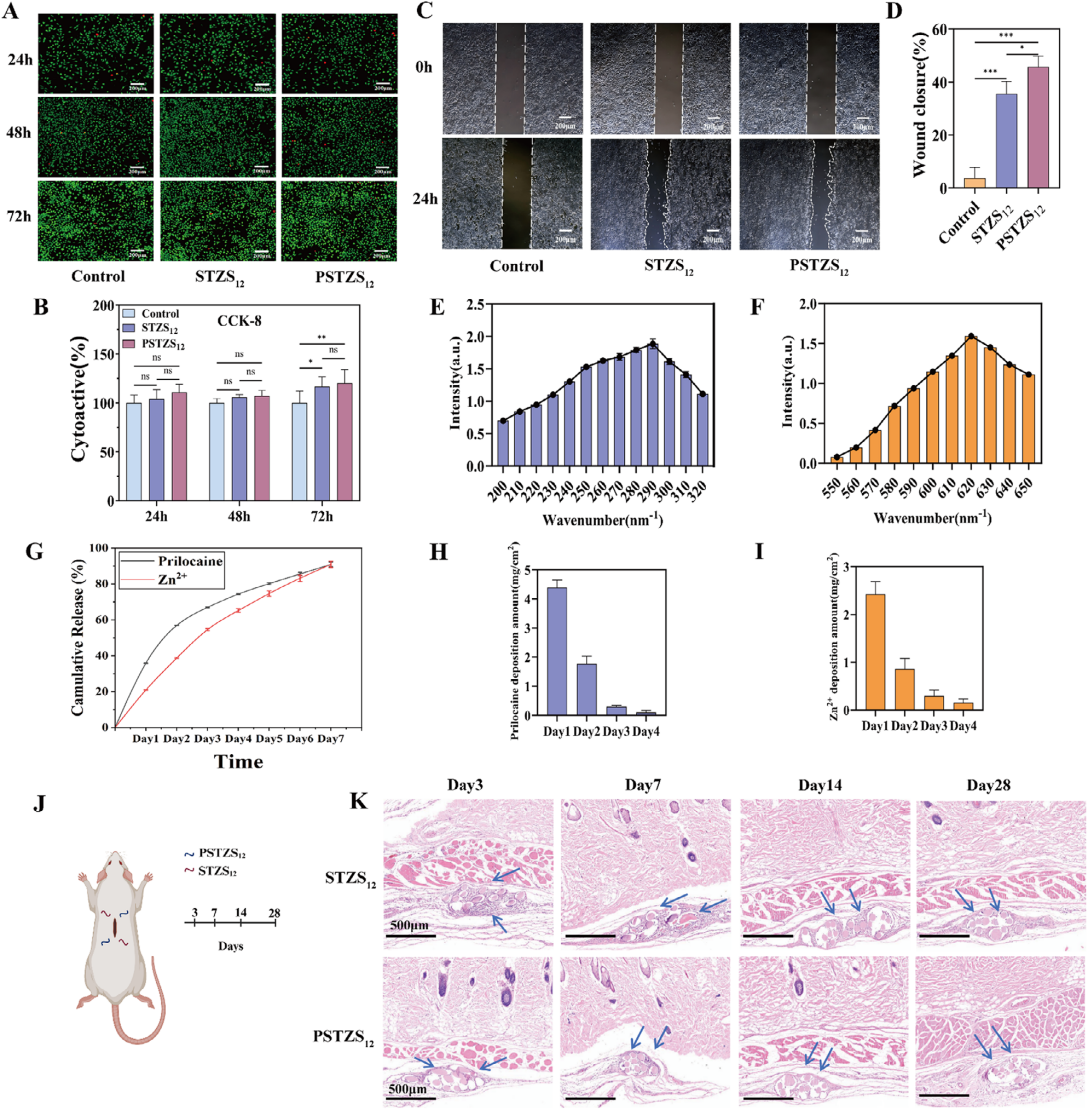
Biocompatibility and cell migration assays. (A). Live and dead fibroblasts were stained using Calcein/PI and CCK‐8 assays, with green indicating live cells and red indicating dead cells. (B). CCK‐8 assay results. (N = 5) (C, D). Fibroblast migration assay and analysis. (N = 3). (E, F). Characteristic UV absorption peaks and wavelengths of prilocaine(E) and zinc ions(F). (N = 5). (G). In vitro drug release of Zn^2^^+^ and Prilocaine from PSTZS_12_. (H, I). Determination of prilocaine and zinc ion deposition in rat dorsal skin incision(N = 6). (J). In vivo biocompatibility evaluation with implantation and sampling schedule for STZS_12_ and PSTZS_12_ (Created in BioRender. Peng, Y. (2026) https://BioRender.com/s84q535). (K). H&E staining of STZS_12_ and PSTZS_12_ implants at 3, 7, 14, and 28 days, with sutures marked by blue arrows. Data are expressed as mean ± standard deviation (SD).

We conducted live/dead staining and CCK‐8 assays to evaluate the cytotoxicity of STZS_12_ and PSTZS_12_ extracts on L929 fibroblasts. Figure 6A shows significant fibroblast proliferation over 24, 48, and 72 hours with extracts, with no differences between STZS_12_, PSTZS_12_, and control groups. The CCK‐8 assay, depicted in Figure 6B, further confirmed that both STZS_12_ and PSTZS_12_ groups sustained high cell viability. These findings indicate that STZS_12_ and PSTZS_12_ are non‐toxic to cells, implying their good biocompatibility.

In Figure 6C,D, the fibroblast migration assay conducted on the 24th hour revealed that both STZS_12_ and PSTZS_12_ effectively facilitated fibroblast migration, which is a key process for wound healing. The wavelength‐specific absorbance maxima of prilocaine and zinc ions were determined spectrophotometrically (Figure 6E,F). Prilocaine exhibited a sharp absorption maximum at 290 nm, while zinc ions yielded a well‐defined peak centred at 620 nm.

To foster comfortable wound healing, we examined the impact of Prilocaine and zinc ions. PSTZS_12_ was placed in a PBS solution, and its optical density (OD) value was measured at various time points, as detailed in Figure 6G. The release rate of Prilocaine was a modest 35.84% on the first day, increasing to 67% after three days, after which the release rate gradually slowed. The zinc ion release started at 21% on the first day, reaching 75% after five days, followed by a gradual deceleration.The more rapid initial release of Prilocaine compared to zinc is due to Prilocaine's positioning in the outer layer of the suture, while zinc is embedded in the inner layer. The low initial release rates for both substances may be attributed to their low solubility in water, a factor that can influence the initial release profile from drug‐eluting materials. This controlled release mechanism is designed to provide a sustained therapeutic effect, aligning with the needs of effective wound management. Our sutures contained ≈ 10.5 mg cm^−1^ prilocaine and ≈ 12.5 mg cm^−1^ zinc. According to Equation 1, we back‐calculated cumulative release from a 1 cm suture: prilocaine, 3.76, 2.23, and 1.04 mg on days 1–3; Zn^2+^, 3.38, 2.25, and 1.59 mg on days 1–3; and prilocaine, 0.78, 0.63, 0.59, and 0.58 mg on days 3–7, while Zn^2+^ release was 1.21, 1.15, 1.13, and 1.08 mg. Recent studies have established effective subcutaneous and topical doses of 10–60 mg for prilocaine [41, 42] and 0.5–2 mmol/L(≈8.25–32.5 mg/kg) for Zinc [43]. All values remained below the established therapeutic and safety thresholds, ensuring no adverse effects on tissues or the organism and confirming their excellent safety profile.

Figure 6H,I demonstrate that within the first 24 h after full‐thickness dorsal incisions were closed with PSTZS_12_ sutures, prilocaine and Zn^2+^ rapidly accumulated in the perilesional tissue to 4.39 and 2.42 mg cm^−2^, respectively—concentrations commensurate with the reported thresholds for sustained analgesia and effective antimicrobial prophylaxis [44, 45]. Subsequently, both agents exhibited mono‐exponential decay kinetics (R^2^ ≥ 0.97) that tracked the proliferative phase of wound repair, implying coupled passive diffusion and active metabolic clearance. By post‐operative Day 4, residual levels had declined to 0.10 mg cm^−2^ (prilocaine) and 0.15 mg cm^−2^ (Zn^2+^), indicating near‐complete elimination after their primary therapeutic functions had been fulfilled.

STZS_12_ and PSTZS_12_ sutures were subcutaneously implanted in rats for 3, 7, 14, and 28 days (Figure 6J). As shown in Figure 6K, both sutures induced minimal inflammation with few macrophages around them after 3 days (shown by blue arrows), which gradually decreased over 14 and 28 days. Minimal inflammation was observed with STZS_12_ and PSTZS_12_. These results suggest good in vivo biocompatibility for both sutures.

**FIGURE 7:**
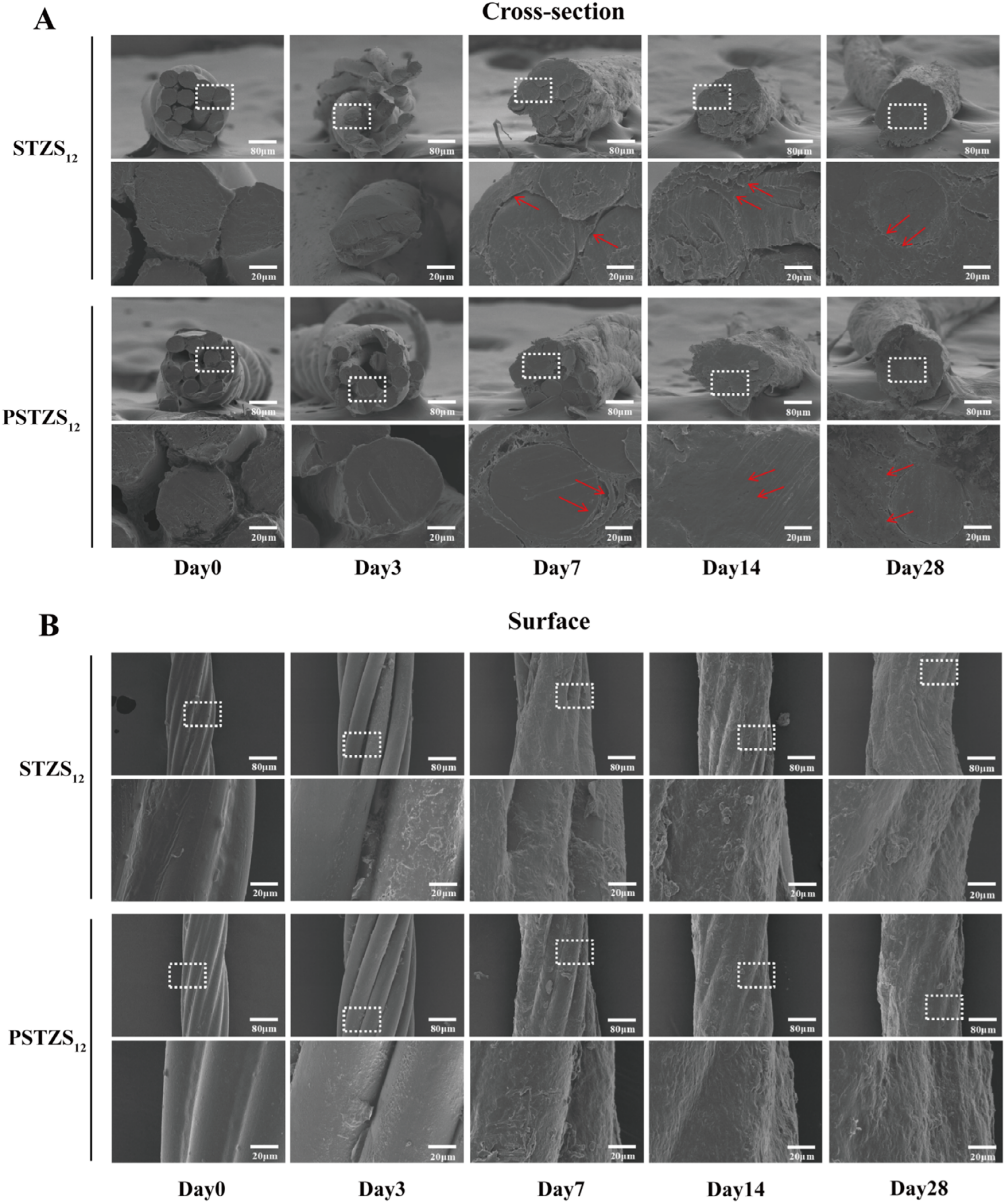
SEM Analysis of STZS_12_ and PSTZS_12_ after Subcutaneous Implantation in Rats. (A). Cross‐sectional view. (B). Surface view.

The remaining STZS_12_ and PSTZS_12_ sutures after 3, 7, 14 and 28 days were carefully retrieved for SEM analysis to scrutinize their morphological evolution. Figure 7A captures the cross‐sectional view, revealing that the internal fiber architecture of both STZS_12_ and PSTZS_12_ sutures initially contained considerable gaps and voids, as also depicted in Figure 7B. Within a brief period of 3 days, the outer SerMA coating commenced degradation, potentially being absorbed by the enveloping tissue.

As the timeline extended to 7, 14, and 28 days, there was a marked reduction in the inter‐fiber pores(shown by red arrows), concurrent with the fibers undergoing mechanical degradation through tissue encapsulation [8]. This encapsulation is believed to stem from the initial degradation that paved the way for tissue infiltration into the STZS_12_ and PSTZS_12_ sutures. Externally, the STZS_12_ and PSTZS_12_ sutures demonstrated a progressive enlargement over time, with their surfaces becoming increasingly smooth and compact. This gradual surface refinement is likely a consequence of tissue integration and the dissipation of torsional stresses through the degradation process.

The smoother and denser surfaces of the sutures over time suggest a synergistic interaction with the surrounding tissue, pointing to a well‐tolerated and biocompatible material that meshes seamlessly with the biological milieu. This harmonious integration signifies the sutures' capacity to engage constructively with the host environment, showcasing their potential for supporting wound healing with minimal irritation or rejection.

### 2.4 In Vitro Antibacterial Assay

**FIGURE 8:**
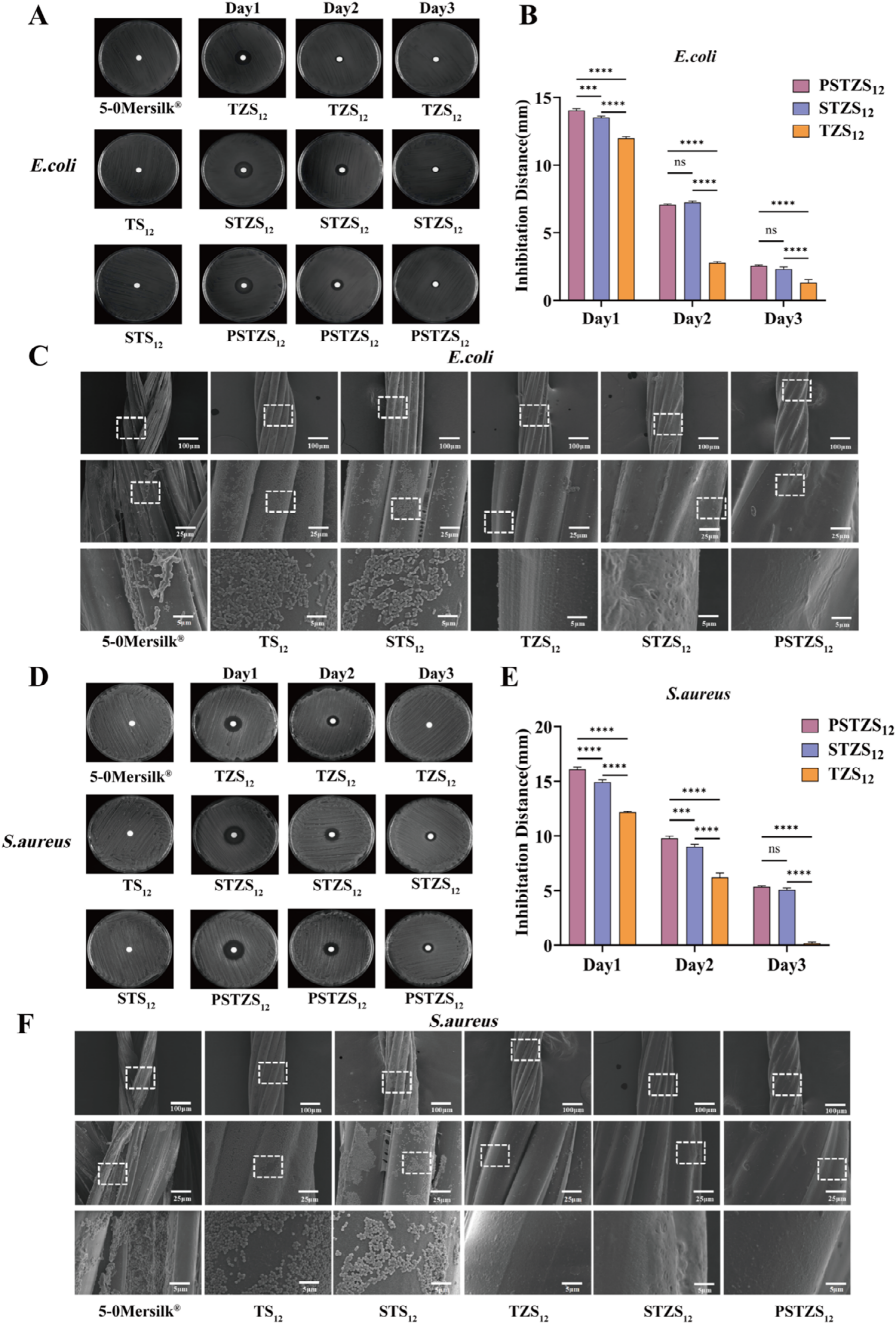
In vitro antibacterial properties. (A). Inhibition zones of 5‐0 Mersilk, TS_12_, STS_12_, TZS_12_, STZS_12_ and PSTZS_12_ on *E. coli* culture plates after 1, 2 and 3 days. (B). Statistical analysis of inhibition distances for TZS_12_, STZS_12_ and PSTZS_12_ on *E. coli* culture plates. (C). SEM analysis of *E. coli* adhesion on 5‐0 Mersilk, TS_12_, STS_12_, TZS_12_, STZS_12_ and PSTZS_12_. (D). Inhibition zones of 5‐0 Mersilk, TS_12_, STS_12_, TZS_12_, STZS_12_ and PSTZS_12_ on *S. aureus* culture plates after 1, 2 and 3 days. (E). Statistical analysis of inhibition distances for TZS_12_, STZS_12_ and PSTZS_12_ on *S. aureus* culture plates. (F). SEM analysis of *S. aureus* adhesion on 5‐0 Mersilk, TS_12_, STS_12_, TZS_12_, STZS_12_ and PSTZS_12_. All experiments were performed in triplicate (N = 3), and data are presented as mean±SD.

The antimicrobial properties of TZS_12_, STZS_12_, and PSTZS_12_ were rigorously evaluated in compliance with the ISO 20645:2004 standard, where an inhibition zone exceeding 10 mm is considered indicative of antimicrobial activity. As depicted in Figure 8A, in contrast to the 5‐0 Mersilk sutures and TS_12_ and STS_12_ extracts, which did not exhibit significant inhibitory circles on *Escherichia coli* (ATCC 25922) agar plates, the extracts from TZS_12_, STZS_12_, and PSTZS_12_ demonstrated substantial antimicrobial effects. On the first day, the inhibitory circles for TZS_12_, STZS_12_, and PSTZS_12_ on *E. coli* plates were measured at 11.98, 13.51, and 14.03 mm, respectively. These values decreased to approximately 2.77, 7.24, and 7.05 mm by the second day, and further reduced to 1.13, 2.31, and 2.54 mm by the third day, illustrating the correlation between the size of the inhibition zone and the concentration of the antimicrobial agents over time.

Figure 8B elucidates that STZS_12_ and PSTZS_12_ exhibit a controlled, sustained release of zinc ions, which contributes to their enduring antimicrobial efficacy. Figure 8C shows a stark contrast in bacterial colonization; *E. coli* was observed to adhere to the 5‐0 Mersilk sutures, TS_12_, and STS_12_, while no such colonization was detected on TZS_12_, STZS_12_, and PSTZS_12_. This observation underscores the persistent antibacterial effect of STZS_12_ and PSTZS_12_ against E. coli.

Figure 8D reflects similar findings with *Staphylococcus aureus* (ATCC 25923), where the 5‐0 Mersilk sutures, TS_12_, and STS_12_ infusions failed to exhibit significant inhibitory circles on agar plates. In stark contrast, extracts from TZS_12_, STZS_12_, and PSTZS_12_ displayed pronounced inhibition. The inhibitory circles on *Staphylococcus aureus* plates for TZS_12_, STZS_12_, and PSTZS_12_ were 12.16, 14.91, and 16.08 mm on the first day, respectively, diminishing to approximately 6.20, 9.01, and 9.78 mm by the second day, and further to 0, 5.06, and 5.33 mm by the third day. This reduction in the inhibition zone over the three days underscores the decreasing concentration of the antimicrobial substances.

Figure 8E demonstrates that STZS_12_ and PSTZS_12_ have a layer of zinc ions that are released slowly over time, maintaining a prolonged antimicrobial effect. Figure 8F reveals that *S. aureus* was observed to colonize and adhere to the 5‐0 Mersilk sutures, TS_12_, and STS_12_, while no bacterial colonization was detected on TZS_12_, STZS_12_, and PSTZS_12_. This finding indicates that STZS_12_ and PSTZS_12_ possess a sustained antibacterial effect against *S. aureus*, offering a significant advantage in preventing wound infections and promoting healing in a controlled and effective manner.

### 2.5 In Vivo Pain Relief From Behavioral Testing to PET/CT ^18^F‐FDG Evaluation

By integrating procaine into our suture, we aim to harness its analgesic benefits for superior management of postoperative pain. This innovative integration of bioactive elements exemplifies our commitment to advancing suture technology for better patient outcomes.

**FIGURE 9:**
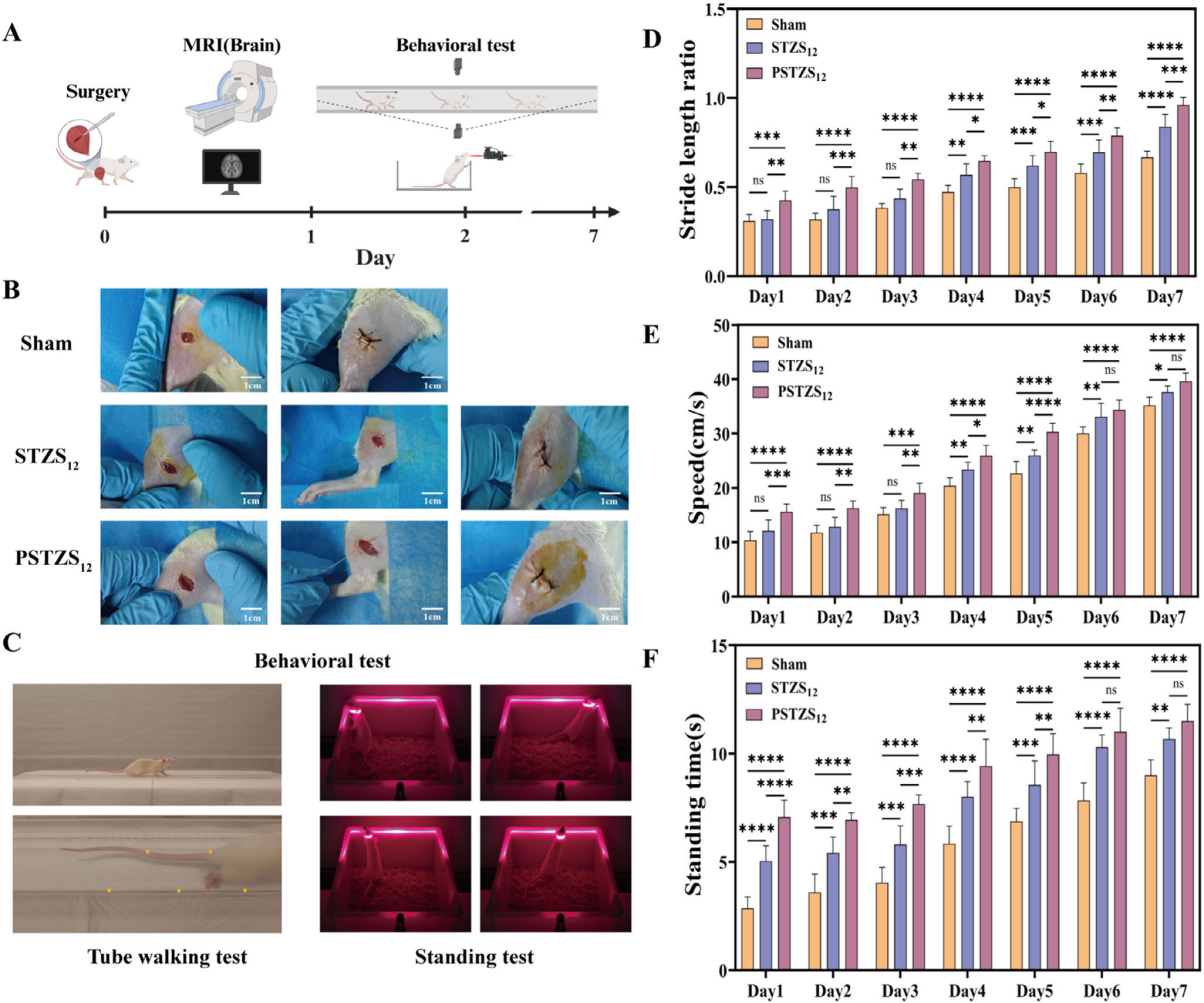
The quadriceps incision model and behavioral assessment tests in rats. (A). Schematic and timeline of experimental behavioral and PET/CT testing of analgesic sutures in the quadriceps muscle (Created in BioRender. Peng, Y. (2026) https://BioRender.com/4wp1jwt). (B). Quadriceps injury suture model. (C). Pipeline walking experiment and standing experiment. (D–F). 7‐day behavioral statistical indexes (including walking stride, speed, and standing time), (D). 7‐day walking stride (surgical group/normal rats), (E). 7‐day walking speed, (F). 7‐day standing time. All experiments were performed in triplicate (N = 6), and data are presented as mean±SD.

The timeline of the animal experiment is shown in Figure 9A. In our quest to assess the efficacy of STZS_12_ and PSTZS_12_ sutures in pain management, we meticulously constructed a pain evaluation model by performing an incision in the left quadriceps muscle of rats, as detailed in Figure 9B. Pain levels were then meticulously evaluated by scrutinizing the rats' movements, encompassing walking speed, stride length, and standing time, all of which are vividly captured in Figure 9C. The underlying principle is that diminished mobility in these animals is indicative of heightened pain levels. Initially, we calculated the stride ratios (surgical rats to normal rats). Figure 9D illustrates that while the ratio increased gradually across all groups, the Sham and STZS_12_ groups exhibited no significant difference in walking stride by the third postoperative day. Conversely, the PSTZS_12_ group demonstrated a markedly larger stride, with no significant divergence observed compared to normal rats by the seventh day.

We proceeded to examine the walking speed of the rats, as depicted in Figure 9E. The PSTZS_12_ group consistently displayed a more rapid speed than both the sham‐operated group and the STZS_12_ group throughout the entire 7‐day observation period. For the initial 3 days post‐surgery, no significant difference in speed was noted between the STZS_12_ and Sham groups. However, by the fourth day, the STZS_12_ group's speed had significantly surpassed that of the Sham group. For instance, after 4 days, the velocities of rats in the sham‐operated, STZS_12_, and PSTZS_12_ groups were 20.4, 23.4, and 25.9 cm/s, respectively.

These findings suggest that 3‐day post‐operation, the analgesic effect of Prilocaine in the PSTZS_12_ group resulted in significantly improved stride and velocity compared to the STZS_12_ group. By the fourth day after surgery, both the PSTZS_12_ and STZS_12_ groups had rapidly entered the proliferative phase, with their walking speeds not significantly differing from days 4 to 7. This is likely attributed to the pro‐angiogenic effects and fibroblast proliferation induced by zinc ions, which expedited the wound healing process.

To conclude our evaluation, we measured the standing time of the rats, noting that a shorter duration of standing typically correlates with more intense pain. Figure 9F demonstrates that the PSTZS_12_ group had a significantly longer standing time compared to the Sham and STZS_12_ groups from day 1 to day 7, suggesting reduced pain levels. Thus, the PSTZS_12_ sutures effectively alleviated pain, underscoring the therapeutic benefits of these sutures in diminishing postoperative discomfort and facilitating a more comfortable recovery process.

The localized, sustained release of Prilocaine in the first three days postoperatively acts to stabilize nerve cell membranes by blocking the ionic currents essential for the generation and conduction of nerve impulses, thereby producing a profound local anesthetic effect [46]. This controlled release provides a gentle buffer against the initial surge of postoperative pain, ensuring a more tranquil experience for the healing process.

As the wound begins to progress from day 4 to 7, the influence of zinc ions becomes particularly significant. These ions stimulate the proliferation of vascular endothelial cells and fibroblasts, thereby accelerating the wound‐healing process. This acceleration not only reduces pain but also enhances the rats' mobility as the healing tissues regain their strength and function more effectively.

This synergistic approach, where Prilocaine offers immediate pain relief and zinc ions bolster the body's innate healing mechanisms, creates a comprehensive strategy for postoperative care. By tackling both the immediate need for pain management and the subsequent requirements for efficient wound healing, this dual‐action strategy not only mitigates pain but also optimizes recovery and mobility, highlighting the sophisticated benefits of these cutting‐edge suture materials in facilitating a smoother and more comfortable healing journey.

**FIGURE 10:**
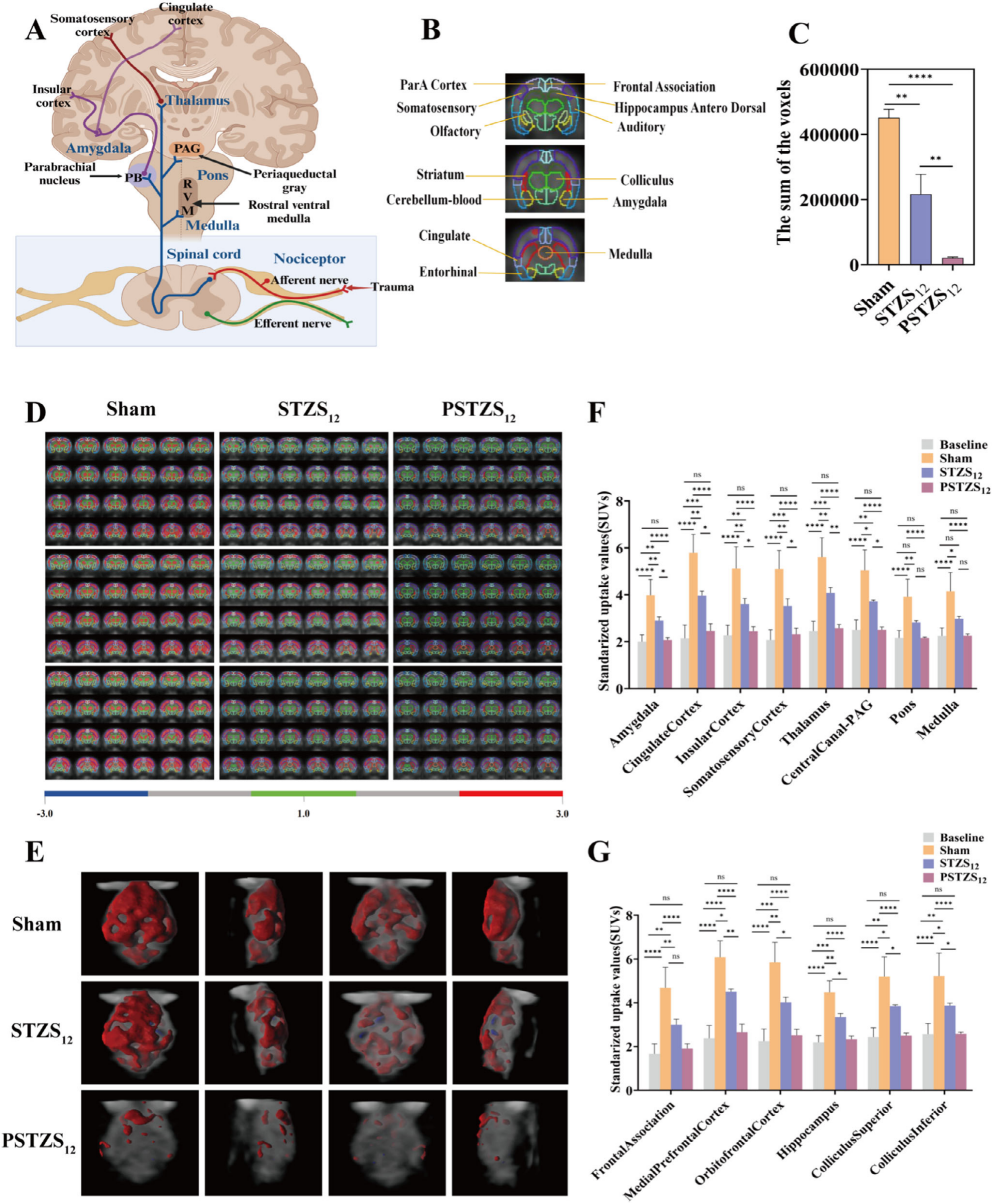
Pain Pathway Anatomy and Brain Activity Analysis. (A). Schematic of the pain pathway anatomy (Created in BioRender. Peng, Y. (2026) https://BioRender.com/om7wblu). (B). Brain region segmentation in CT scans using the PMOD rat model. (C). Total voxel count in brain regions with Z‐scores>3 (N = 3). (D). PET/CT imaging of brain regions with |Z|>3 (red: Z>3; blue: Z←3). (E). 3D^18^F‐FDG images of brain regions with |Z|>3, viewed from the top, left, bottom and right. (F). SUV measurements of ^18^F‐FDG uptake in primary pain conduction‐related regions. (G). SUV measurements of ^18^F‐FDG uptake in pain modulation‐related regions. (Brain regions with Z>3 are highlighted in red, representing activated areas, while those with Z←3 are shown in blue, indicating inhibited regions.) (Data presented as mean±SD, N = 3).

Figure 10A illustrates the complex pathway of pain signal transmission in the nervous system. When exposed to external injury, primary nociceptor afferent fibers rapidly send pain signals to projection neurons in the spinal cord's dorsal horn. These neurons then activate ascending pathways that relay pain information to the thalamus and brainstem. The thalamus plays a crucial role in pinpointing the location and intensity of the painful stimulus. Meanwhile, other projection neurons enhance the pain experience by connecting to perceptual areas. They extend to the brainstem (especially the parabrachial nucleus) and the amygdala, which are central to the emotional response to pain. These neurons are also linked to the insular and cingulate cortices, which are vital for the sensory and emotional aspects of pain perception [47, 48].

Ascending pain signals are conveyed and regulated by neurons in the medullary gray matter, which project toward the midbrain aqueduct. Research has indicated that the cingulate cortex, insular cortex, prefrontal cortex, somatosensory cortex, thalamus, and orbital cortex are key regions involved in pain perception [49]. In contrast, the gray matter of the medulla oblongata, the brainstem, and the midbrain aqueduct are chiefly involved in the conduction and feedback regulation of pain signals [50].

Moreover, pain can stimulate the activation of parasympathetic nerves, instigating neuroprotective mechanisms. These mechanisms are facilitated through medullary vagal neurons that project to the thalamus, hippocampus, cerebral cortex, and brainstem. They promote the secretion of various neurotrophic factors, which modulate cerebrovascular blood flow and thereby safeguard the nervous system [51].

Initially, 12 healthy rats underwent baseline PET/CT scans to measure the ^18^F‐FDG uptake levels in their brains. Following surgery, on the first postoperative day, three rats from each group—STZS_12_, PSTZS_12_, and sham‐operated—were subjected to ^18^F‐FDG imaging. To account for differences in body weight among the rats and ensure more precise calibration of ^18^F‐FDG uptake, standardized uptake values (SUV) were carefully calculated.

The Z‐score serves as an alternative metric to delineate the variance between the experimental cohort and its initial state [52, 53]. We delineated the pertinent brain regions with |Z|>3 in the rat brain tissue, relying on the zonal localization of the rat brain tissue as ascertained by the PMOD method across three representative CT sections shown in Figure 10B. Figure 10C illustrates the aggregate SUVs for ^18^F‐FDG imaging, focusing on areas exceeding the |Z|>3 criterion. Notably, both ^18^F‐FDG uptake and somatostatin levels were markedly reduced in the PSTZS_12_ group relative to the sham‐operated and STZS_12_ groups.

In Figure 10D, brain regions with Z > 3 are highlighted in red, representing activated areas, while those with Z < ‐3 are shown in blue, indicating inhibited regions. It is noteworthy that the PSTZS_12_ and STZS_12_ groups exhibited significantly fewer red‐marked (activated) areas compared to the sham‐operated group, which had extensive red regions. Moreover, the PSTZS_12_ group showed an even greater reduction in activated regions, suggesting a more pronounced effect on brain activity.

In Figure 10E and Video S4, we conducted a meticulous comparison of ^18^F‐FDG metabolism levels across the Sham, STZS_12_, and PSTZS_12_ groups, utilizing 3D modelling with a significance threshold of |Z|>3. This sophisticated method enabled us to identify regions with substantial metabolic activity. Figure 10F,G reveal that the PSTZS_12_ group demonstrated a significantly lower SUV, closely approximating baseline levels in the majority of pain‐related regions, and were statistically indistinguishable from baseline. This suggests that the PSTZS_12_ group exhibited a subdued metabolic response in these areas, indicative of effective pain management. Conversely, the Sham group displayed a pronounced increase in pain‐related brain regions compared to the PSTZS_12_ group and the baseline group, signifying a more rapid metabolic rate. The increased metabolic activity could indicate more vigorous signal processing and protein synthesis, pointing toward a heightened sensitivity to painful stimuli [54].

The STZS_12_ group showed no significant difference in SUV compared to the baseline group in the brainstem and medulla oblongata. This lack of significant change might be attributed to the activation of the periaqueductal gray (PAG) and the rostral ventromedial medulla (RVM). These regions regulate pain signaling via a negative feedback loop, which can limit the propagation of pain signals and help maintain metabolic stability in the affected areas [55]. This suggests that the PSTZS_12_ sutures' analgesic properties could be the result of intricate neurobiological pathways that alter the central nervous system's pain processing, indicating a nuanced approach to managing pain and underscoring the therapeutic potential of these advanced suture materials.

### 2.6 Rat Dorsal Skin Suturing Experiment and Wound Healing

**FIGURE 11:**
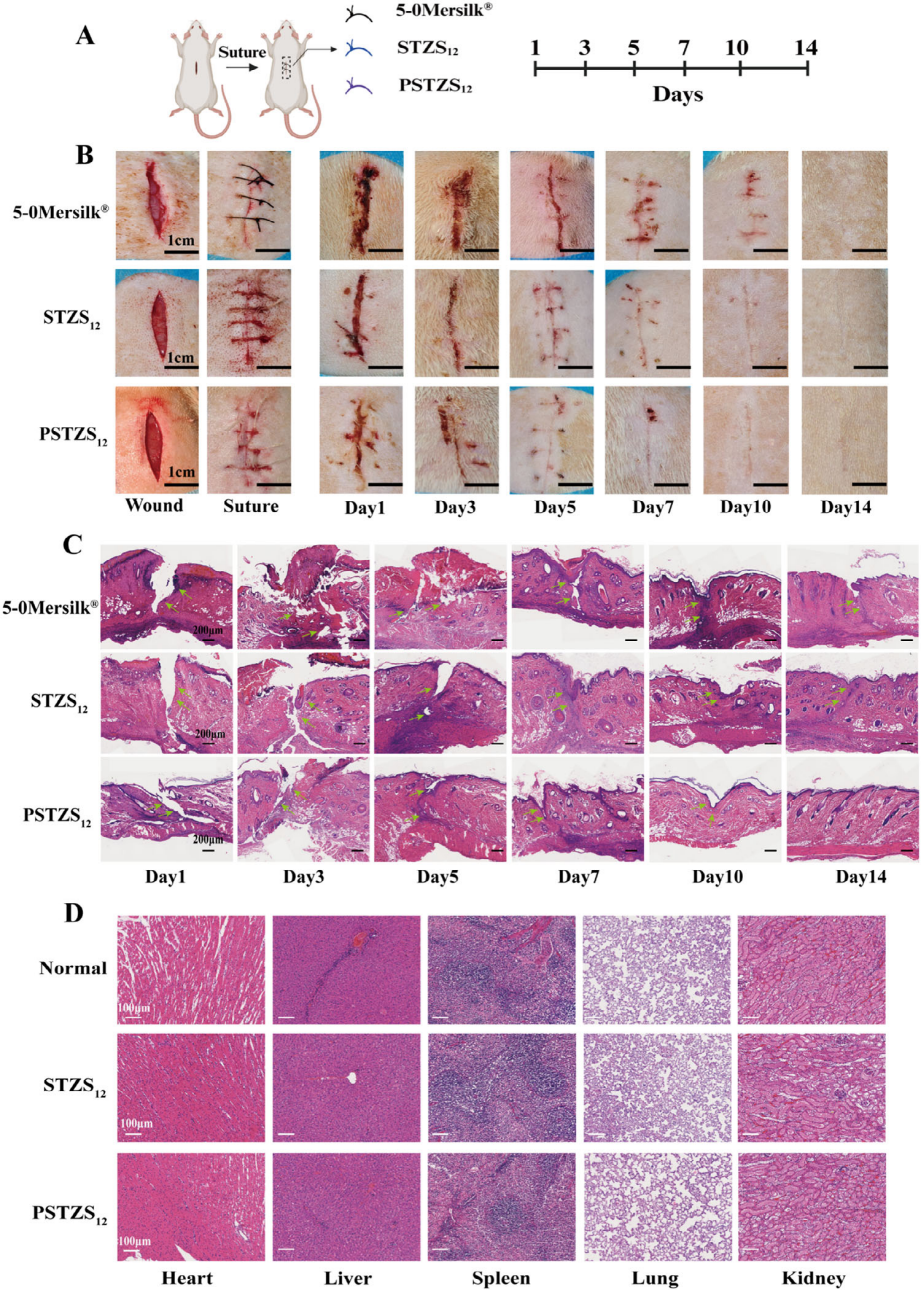
Rat dorsal skin suturing experiment and wound healing. (A). Schematic of rat dorsal skin suturing experiment (Created in BioRender. Peng, Y. (2026) https://BioRender.com/d37z769). (B). Representative photos of the wound healing process in rats sutured with 5‐0Mersilk, STZS_12_ and PSTZS_12._ (Day 1, 3, 5, 7, 10 and 14). (C). H&E staining of rat dorsal skin tissue at various time points representing the healing process. (D). Organ staining of rats with subcutaneously implanted STZS_12_ and PSTZS_12_ sutures after 28 days. (Heart, Liver, Spleen, Lung, Kidney).

To explore the wound‐healing advantages of PSTZS_12_, we conducted a study with 2 cm×2 mm incisions on rat backs, sutured using 5‐0 Mersilk, STZS_12_, and PSTZS_12_. We monitored the healing process on postoperative days 1, 3, 5, 7, 10, and 14(in Figure 11A). As the Figure 11B showed that the STZS_12_ and PSTZS_12_ groups had quicker wound closure and less inflammation than the 5‐0 Mersilk group. In the first three days after surgery, all groups formed scabs, a common early sign of healing. By day 5, the wounds in the STZS_12_ and PSTZS_12_ groups had entered the proliferative phase and closed, while the 5‐0 Mersilk group still had significant scabbing and inflammation. This was likely due to the high tension of the 5‐0 Mersilk suture, which can cause tissue damage, bleeding, and inflammation. On day 7 and 10, the 5‐0 Mersilk group still had some inflammation, while the STZS_12_ and PSTZS_12_ groups showed good healing. By day 14, the healing process was complete in all groups.

Histological analysis with hematoxylin and eosin (H&E) staining (Figure 11C) revealed that on days 1 and 3, scabs began to cover the incision edges (with green arrows indicating the direction of the edges). By day 5, the 5‐0 Mersilk group had extensive scab formation due to tissue damage from the high‐tension suture, while the wounds in the STZS_12_ and PSTZS_12_ groups began to close, with PSTZS_12_ showing a faster rate. By day 7, the epidermis in the 5‐0 Mersilk group had healed via scab, but the dermal layer still had an open incision, whereas the wounds in the STZS_12_ and PSTZS_12_ groups were fully closed. On day 10, the incision edges in the 5‐0 Mersilk group had closed with many inflammatory cells aiding repair. By day 14, the skin in the PSTZS_12_ and STZS_12_ groups was smooth, while the 5‐0 Mersilk group still had noticeable indentations.

To verify the biocompatibility of STZS_12_ and PSTZS_12_, we examined the hearts, livers, spleens, lungs, and kidneys of rats after 28 days of subcutaneous implantation. As the Figure 11D showed no significant toxic effects were observed in these organs, which maintained normal structure and function, similar to those of normal rats.

### 2.7 Histological Analysis of Postoperative Wounds in Rats

**FIGURE 12:**
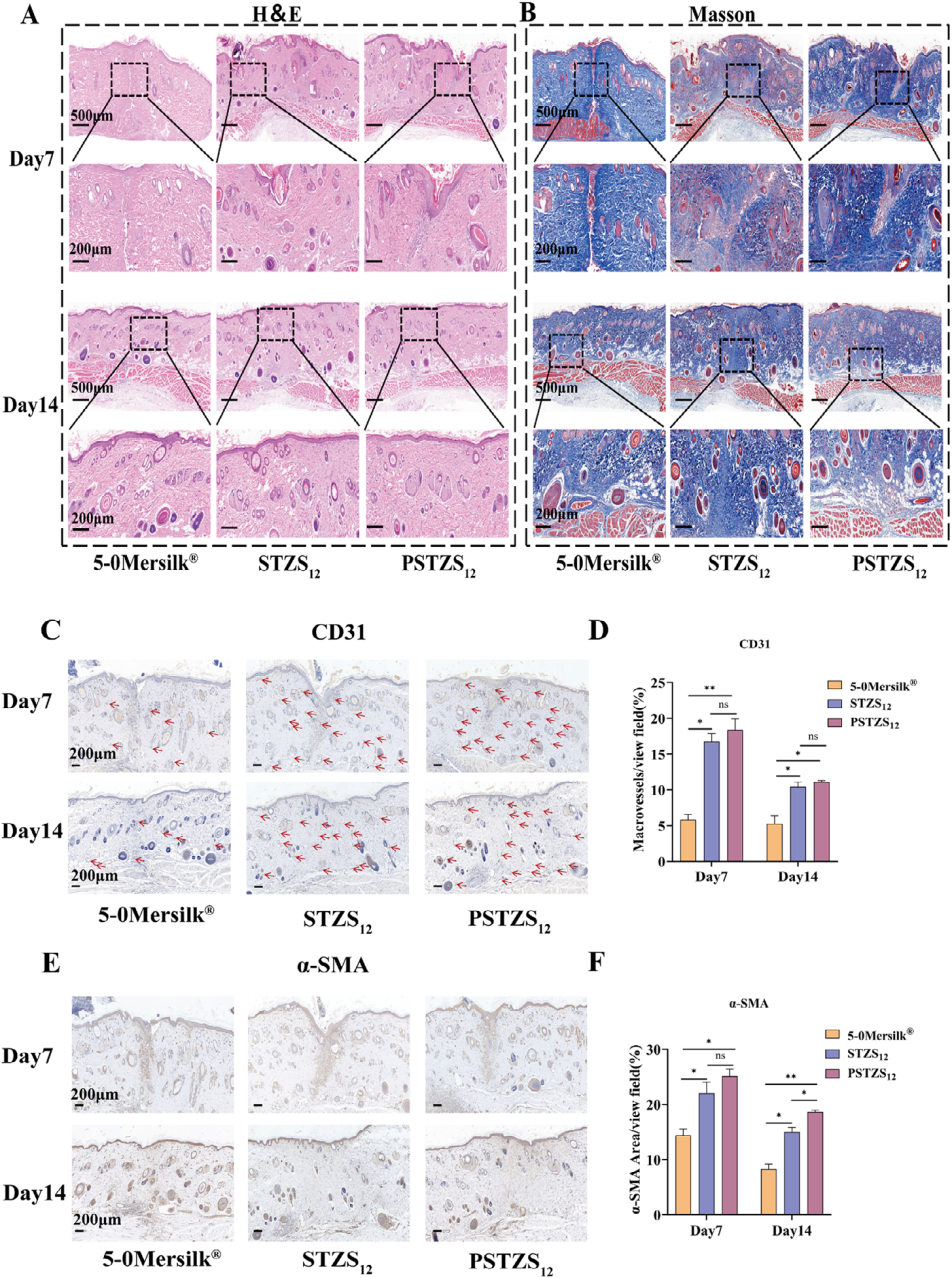
Histological analysis of postoperative wounds in rats. (A, B). H&E and Masson's trichrome staining of incisions at Day 7 and 14. (C). CD31 staining of incisions at Day 7 and 14. (D). Statistical analysis of CD31 in incision tissues on Days 7 and 14. (E). α‐SMA staining of incisions at Day 7 and 14. (F). Statistical analysis of α‐SMA in incision tissues on Days7 and 14. (All data presented as mean±SD, N = 3).

In our research, we closed the dorsal incisions of rats with 5‐0Mersilk, STZS_12_, and PSTZS_12_ sutures. To further investigate the reparative effects of STZS_12_ and PSTZS_12_, the skin samples were harvested on days 7 and 14 for Hematoxylin and Eosin (HE) and Masson's trichrome staining, as Figure 12A,B shown. On day 7, which is the remodeling phase, the STZS_12_ and PSTZS_12_ groups showed a significantly faster wound closure rate than the Mersilk group. Masson's trichrome staining indicated that these groups also had a greater accumulation of collagen fibers at the wound site than the Mersilk group. By day 14, all wounds had healed completely. However, the STZS_12_ and PSTZS_12_ groups demonstrated more intense angiogenesis and a larger amount of collagen fibers surrounding the blood vessels, indicating that these sutures might enhance collagen deposition and angiogenesis.

Additionally, α‐SMA/CD31 immunohistochemical staining was conducted on skin tissues harvested on days 7 and 14, as depicted in Figure 12C. The STZS_12_ and PSTZS_12_ groups exhibited markedly greater endothelial cell proliferation than the Mersilk group on both assessment days. On day 7, a profusion of neovascularized capillaries was evident at the incision margins (indicated by red arrows), and these persisted on day 14 despite complete wound closure. Figure 12E illustrates a significant deposition of smooth muscle cells around the incisions in the STZS_12_ and PSTZS_12_ groups on day 7, actively participating in tissue repair. By day 14, PSTZS_12_, STZS_12_ continued to stimulate smooth muscle cell proliferation and contribute to wound healing.

Figure 12D,F display the quantification of CD31 and α‐SMA in rat skin tissues. The results indicate that the levels of CD31 and α‐SMA were elevated in the skin tissues of the STZS_12_ and PSTZS_12_ groups compared to the Mersilk group on both day 7 and day 14. The levels were notably higher on day 7 compared to day 14, as the wound was in the remodeling phase on day 7, characterized by abundant collagen and active migration of vascular endothelium to the wound site to facilitate healing.

In summary, our study demonstrates that STZS_12_ and PSTZS_12_ significantly enhance wound healing, collagen deposition, angiogenesis, and endothelial cell proliferation in rat dorsal incisions. Compared to the 5‐0 Mersilk suture, these materials exhibit lower tissue tension and are less likely to cut into tissues, thereby accelerating the wound‐healing process. These findings strongly suggest that STZS_12_ and PSTZS_12_ could be effectively applied in clinical wound care.

## 3 Discussion

Despite the widespread adoption of hydrogel fibers in medicine, the inherent strength–toughness trade‐off remains a fundamental bottleneck in the development of high‐performance fibrous biomaterials [56]. Conventional reinforcement strategies—including mechanical drawing, metal‐ion cross‐linking, chemical coordination, and carbon nanomaterial incorporation—only partially enhance mechanical indices and fall short of the multifaceted demands of advanced surgical applications [57, 58, 59].

Utilizing wet spinning, we crafted regenerated silk protein hydrogel fibers. Lithium bromide played a pivotal role in dissolving the complex structure of natural silk fibroin by breaking the hydrogen bonds and van der Waals forces inherent in the β‐sheet assemblies [18]. Post this dissolution, the aqueous silk protein solution was subjected to dialysis to eliminate ionic salts, paving the way for the formation of regenerated silk fibroin through freeze‐drying. Formic acid then dissolved the regenerated silk fibroin proteins [60], and a 2.5% Zn^2+^ methanol solidification bath was employed to induce rapid formation of β‐sheet folded nanocrystals within the regenerated silk proteins [61]. These nanocrystals are crucial for the fibers' stability and for enhancing their mechanical properties. Zinc ions in the coagulation bath formed ionic interactions with free serine residues, enhancing the fibers' mechanical properties [30]. The application of pull force during drawing induced molecular ordering and alignment in the fibers, resulting in significantly enhanced mechanical properties [37].

Here, we report a robust and tunable regenerated silk fibroin fiber fabricated via an exceptionally facile and scalable route. The resulting fibers exhibit an ultimate tensile strength of 804 MPa and a Young's modulus of 43 GPa, underpinned by a high degree of crystallinity and a β‐sheet‐rich secondary structure. This work establishes a new technological paradigm and theoretical framework for next‐generation biomedical devices—including high‐strength wearable biosensors, mesh scaffolds, biological patches, surgical sutures, and even artificial tendons.

Post‐operative bacterial infection and pain remain the principal complications that compromise wound healing and functional recovery [62, 63]. Early intervention is therefore pivotal for shortening convalescence and mitigating healthcare burdens. Owing to its broad‐spectrum antimicrobial efficacy and pro‐angiogenic capacity, Zn^2+^ has been demonstrated to markedly suppress bacterial colonization and to accelerate tissue regeneration [27]. Prilocaine, a first‐line amide‐type local anaesthetic that blocks voltage‐gated Na^+^ channels, offers rapid onset and a wide therapeutic index, thereby providing effective relief of acute post‐operative pain [64]. In this study, we engineered a multifunctional, silk‐protein‐based hydrogel suture (PSTZS_12_) integrating antibacterial, analgesic, and wound‐healing functionalities. Compared with the clinically prevalent Mersilk, PSTZS_12_ exhibits superior mechanical properties. The incorporation of Zn^2+^ substantially reduces the adhesion and proliferation of *Escherichia coli* and *Staphylococcus aureus*, while remaining fully biocompatible at physiological concentrations. Furthermore, Zn^2+^ attenuates the inflammatory cascade and diminishes foreign‐body reactions, thereby fostering micro‐environmental homeostasis and facilitating tissue regeneration.

Nociceptive signals evoked by tissue injury ascend through a hierarchical peripheral‐to‐central pathway and ultimately reach the cerebral cortex to elicit pain perception [65]. In addition to observing behavioral signs, we have adopted a more objective method by investigating changes in brain regions that respond to pain. Fluorodeoxyglucose (FDG), a glucose analogue, is preferentially absorbed by cells with high metabolic activity. ^18^F‐FDG, incorporating the radioactive isotope ^18^F, serves as a tracer detectable in PET/CT scans [66]. As it circulates in the bloodstream, it illuminates brain areas with elevated activity, indicating higher glucose metabolism. The intensity of ^18^F‐FDG uptake in these regions directly reflects metabolic activity, with stronger signals suggesting increased glucose utilization and faster metabolism [67]. This ^18^F‐FDG imaging technique has become essential for studying cerebral and spinal responses to pain [68].

Behavioral analyses revealed that PSTZS_12_ significantly improved post‐operative locomotor performance in rats. Consistent with these findings, PET‐CT demonstrated a pronounced reduction in glucose metabolism across fourteen pain‐associated brain regions—including the anterior cingulate cortex, insula, thalamus, and nucleus accumbens—on post‐operative day 1 (P < 0.01), coinciding with enhanced mobility. Mechanistically, the integrated prilocaine likely exerts its analgesic effect by blocking voltage‐gated Na^+^ channels in primary afferent fibers, thereby suppressing action potential propagation and affording dual peripheral and central antinociception.

The wound healing process is intricate, involving several distinct stages: the hemostasis phase (lasting about 1 day), the inflammatory phase (1–3 days), the proliferative phase (3‐7 days), and the remodeling phase (occurring after 7 days) [69]. Comprehensive assessments demonstrate that PSTZS_12_ possesses outstanding biocompatibility. Relative to the clinically prevalent Mersilk sutures, PSTZS_12_ markedly suppresses local inflammatory cascades, accelerates wound closure, and abbreviates the inflammatory phase, thereby exhibiting superior tissue‐regenerative efficacy.

The pro‐angiogenic impact of zinc ions is especially beneficial, as it fosters the growth of new blood vessels critical for tissue repair and the supply of nutrients to the healing area [27, 70, 71]. Moreover, the promotion of fibroblast proliferation by zinc ions aids in the synthesis of the extracellular matrix, which is vital for wound closure and tissue regeneration []. Histological assessment further confirmed that PSTZS_12_ markedly attenuated local inflammatory infiltration, accelerated wound closure, and promoted the orderly deposition of collagen fibers compared with conventional clinical sutures. Immuno‐histochemical staining revealed up‐regulated CD31 and α‐SMA expression, indicative of early endothelialization and neovascularization.

In conclusion, this study pioneers the fabrication of a high‐strength silk fibroin hydrogel fiber, establishing a structural paradigm for next‐generation surgical devices. Leveraging this foundation, we have engineered a core–shell, multifunctional silk fibroin hydrogel suture that exhibits superior biocompatibility while concurrently integrating antibacterial, analgesic, and pro‐regenerative functionalities. Collectively, these findings provide compelling evidence that PSTZS_12_ facilitates rapid, painless, and infection‐free post‐operative wound healing. Nevertheless, this study has the following limitations: (i) the specific molecular mechanisms underlying PSTZS_12_‐mediated regulation of the wound microenvironment (e.g., key signaling pathways and cytokine networks) remain to be elucidated; (ii) an infected wound animal model has yet to be established to systematically validate its anti‐infection and pro‐healing efficacy; and (iii) quantitative evaluation of the in vivo degradation kinetics and bioabsorption period of PSTZS_12_ is currently lacking. These deficiencies will be addressed in subsequent studies.

## 4 Materials and Methods

### 4.1 Preparation of Silk Fibroin and the Regenerated Silk Fibroin Fiber Loaded With Zinc Ions

In our procedure, 35 g of domestic silkworms are cleaned and dried, then subjected to degumming by boiling in a Na_2_CO_3_ solution (prepared with 30 g Na_2_CO_3_ for every 14 L of deionized water (Sinopharm Group Chemical Reagent Co. Ltd.). Post‐degumming, the silkworms are dissolved in a 9.3 mol/L LiBr_2_ solution (Shanghai Marel Biochemical Technology Co., Ltd.) and stirred at 60°C for 4–6 hours. Subsequently, the solution undergoes dialysis with a 3500 MWCO membrane (Shanghai Yuanye Biotechnology Co., Ltd) for 2–3 days. The supernatant is separated via centrifugation at 5000 r/min. The resulting silk fibroin protein solution is then processed through lyophilization in a freeze‐dryer. This technique quickly freezes the solution and decreases the pressure to facilitate the sublimation of the ice, yielding a dense silk fibroin protein scaffold. Lastly, the scaffold is kept at – 80°C to maintain its integrity [72].

We began by dissolving the silk fibroin (SF) scaffold in a formic acid solution (provided by Sinopharm Chemical Reagent Co., Ltd.) to prepare the spinning solution required for wet‐spinning. The coagulation bath was made of methanol (Shanghai Titan Technology Co., Ltd.). Using a microfluidic pump (LSPO2‐3B, Baoding Ditron Electronic Technology Co., Ltd.), the spinning solution was extruded through a 200 µm hose at 0.5–1 ml/min into the coagulation bath. The nascent fibers were solidified in the bath for 10 min, then collected and drawn at 200 mm/min using a mechanical tensile apparatus (WDW‐1, Changzhou Sanfeng Instrument Technology Co., Ltd.) (Video S5). After drawing, the fibers were immersed in a ethanol solution to reinforce them. RSF‐Zn^2+^ crosslinked fibers need to be prepared in a coagulation bath and recrystallization bath containing zinc ions, and the other steps are the same as above. To enhance the antibacterial properties of the fibers, they were fixed in a ZnCl_2_ dH_2_O solution (Video S2).

### 4.2 Preparation of SerMA and Regenerated Silk Protein Bionic Hydrogel Sutures

6 g of commercial sericin powder (Shanghai Macklin Biochemical Technology Co. Ltd.) was weighed, and dissolved in 30 mL pH 9.5 phosphate buffer (Beyotime Biological Reagent Co. Ltd in Shanghai, China) at 35°C, then added 3.6 g methacrylated anhydride (J&K Scientific Ltd in China). This mixture reacted for 12–72 hours at 50°C with stirring. The SerMA solution is dialyzed using a 3500 MWCO membrane for 2 – 3 days, then lyophilized to yield a concentrated powder. Weigh 0.5 g of SerMA powder dissolved in 10 ml of deionized water. Next, 1 g Irgacure 2959 (Titan Technology Co., Ltd, Shanghai, China) is dispersed in 100 mL dH_2_O and mixed with the SerMA solution in a 1000:1 – 100:1 ratio. The combined solution is exposed to 365 nm UV light for 1 min to form a SerMA hydrogel [39].

We extracted multiple strands of zinc – enhanced regenerated silk fibroin fibers and twisted them into sutures. These sutures were immersed in a 5% Prilocaine(Changchun Jinsai Pharmaceutical Co. Ltd.) containing SerMA solution for 2 min to incorporate the local anesthetic [40]. After retrieval, the sutures were exposed to 365 nm UV light for 1 min for photocross‐linking of SerMA. This step created a strong hydrogel structure in the sutures, enhancing their mechanical strength and enabling sustained Prilocaine release for consistent analgesia at the wound site, promoting patient comfort and healing.

### 4.3 Characterisation and Physicochemical Properties of Fibers

RSF, RSF (drawing), RSF‐Zn^2+^ crosslinked, and RSF‐Zn^2+^ crosslinked (drawing) samples were freeze‐dried at −70°C, 1 Pa following initial cooling to −20°C and −80°C. These were then readied for SEM (Hitachi Co. Ltd.) observation, with accurate cross‐sections cut and placed for optimal internal structure viewing. The mechanical properties of fibers coagulated in methanol and 2.5% Zn^2+^ methanol baths were compared using a precision tester. Samples had a 50 mm initial length, and the lower grip moved at 20 mm/min until fiber failure. A force transducer captured data and generated stress‐strain curves, allowing us to record the breaking force and compute the maximum strain.

2 mg of each silk fibroin fiber sample was weighed, combined with 200 mg KBr, milled, and pressed into discs for FTIR analysis(Nicolet is10, Thermo Fisher Scientific (China) Co., Ltd). The scan spanned 500–4000 nm at 32 scans/s [34]. ATR‐FTIR was also employed to analyze SF(raw), SerMA, and Prilocaine under matching settings. Crystallinity was assessed using an X‐ray diffractometer (D2 PHASER A26‐X1‐A2E0B2A0, Bruker AXS GmbH, Germany) at 45 kV and 30 mA with Cu – Kα radiation. Samples were scanned across a 2θ range of 5°–60° in 0.02° steps at a 1.5406 Å wavelength [73]. OMNIC (Thermo Fisher Scientific (China) Co., Ltd.) software processed the FTIR data, and amide I peaks were deconvoluted and refined with PeakFit 4.12(Shanghai Cabe Information Technology Co., Ltd.). Data were organized and visualized as bar graphs in GraphPad 10. XRD data underwent smoothing, deconvolution, and analysis in Origin 2022 (OriginLab Co. Ltd.), with findings presented as histograms in GraphPad 10 (Dotmatics, Inc., USA).

Wide‐angle X‐ray scattering (WAXS) measurements were performed on a XEUSS 3.0 instrument (Xenocs SAS, Inc., France) coupled with a Pilatus 100 K detector (Dectris, Inc., Switzerland) and a Cu micro‐focus source (Genix 3D) providing monochromatic X‐ray radiation (λ = 0.15418 nm, E = 8 keV). The sample‐to‐detector distance was fixed at 31.8 mm, and each exposure was collected for 300 s. 2D scattering patterns were azimuthally integrated using FiT‐2D software to generate 1D intensity profiles. Measurements were performed under vacuum, eliminating the need for air‐scatter background subtraction [74]. Use Python to perform peak deconvolution fitting on a 2D curve and calculate the degree of orientation.

### 4.4 Characterisation of Sutures

#### 4.4.1 Electron Microscopic and Mechanical Characterisation of Sutures

Samples were frozen at −80°C and then freeze‐dried in a pre‐cooled lyophilizer until reaching −70°C and 1 Pa, indicating complete lyophilization, before being set aside for further analysis. Cross‐sections were precisely crafted with a sharp scalpel and positioned perpendicularly on a viewing plate, affixed to vertical beams and oriented horizontally upward for easy observation, which was crucial for accurate measurement and detailed examination of the samples' internal structure. The diameters of the cross‐sections were measured thrice by Image J (National Institutes of Health, USA).

Additionally, we undertook a thorough examination of the mechanical properties of an array of suture configurations, including 12 strands with varying degrees of twist, such as STZS_12_, as well as the TS_6_, TZS_6_, STZS_6_, TS_12_, TZS_12_, 5‐0 Mersilk, and PSTZS_12_ using a mechanical tester. The initial length of each suture was set to 100 mm, and a tensile force was applied at 20 mm/min until the suture broke. The breaking force and maximum strain were recorded to assess the sutures' suitability for surgical applications. To assess the mechanical performance of 5‐0 Mersilk, STZS_12_, and PSTZS_12_ under high‐humidity conditions, the sutures were immersed in PBS for 1 min and subsequently tested at 80% relative humidity using the aforementioned protocol.

#### 4.4.2 Physicochemical Properties of Sutures (FTIR, EDS)

We prepared 2 mg samples of raw silk fibroin, SerMA, Prilocaine, and various suture variants, added 200 mg KBr, ground and pressed them into tablets. These samples were then scanned by FTIR across 500–4000 nm at 32 times per second to obtain peak spectrum data for analyzing chemical composition and molecular structure.

For elemental analysis, the sutures were cut and attached to a stage for EDS (Hitachi Co. Ltd.) analysis, scanning for carbon (C), oxygen (O), nitrogen (N), and zinc (Zn) at 15 kV and 45 mA.

#### 4.4.3 Drug Release Experiments and Bio‐Compatibility Experiments

To accurately determine the maximum UV absorption wavelengths of prilocaine and zinc ions, standard solutions at 0.1 g mL^−1^were prepared and subjected to full‐spectrum scanning from 200 to 720 nm. The resulting UV absorption spectra were recorded and the wavelengths corresponding to the absorption maxima were identified (Universal Hood III, Burroughs, Inc., USA). Prilocaine exhibited a maximum absorbance at 290 nm, whereas zinc ions displayed a peak absorbance at 620 nm.

To assess the release kinetics of Prilocaine and Zn^2+^ from PSTZS_12_ sutures, two 40 cm segments were immersed in 5 mL PBS at 37°C. Over 7 days, 2 mL samples were collected daily and replaced with 2ml fresh PBS [40]. Absorption peaks for Zn^2+^ and Prilocaine were at 620 nm and 290 nm respectively, measured using a gel analyzer. Standard solutions were used to create a standard curve, which helped calculate the daily drug release concentration based on sample OD values. By the dry weight method, it was obtained that PSTZS_12_ loaded 10.5 mg of Prilocaine and 12.5 mg of Zn^2+^ per centimeter.

(1) $$\begin{array}{ccc} & & \mathrm{Cumulative}\;\mathrm{release}\;\mathrm{percentage}\;\left(\%\right)\;\\ & & \;=\frac{5C_{x}+2C_{x-1}+\cdots +2C_{1}}{T}\times 100\% \end{array}$$

Cx (mg/ml) denotes the drug concentration released after x days, while T (mg/ml) indicates the total drug loading.

Using Equation [75] 1, we calculated the cumulative percentage release of Prilocaine and Zn^2+^ over 7 days, gaining insights into the sustained release kinetics of PSTZS_12_ sutures and their potential therapeutic efficacy and duration in clinical use.

To test the biocompatibility of STZS_12_ and PSTZS_12_ sutures, we prepared extracts following ISO10993‐12. Sterilized samples were combined with DMEM, ultrasonicated, and incubated, then filtered. L929 fibroblasts were seeded in a 96‐well plate, treated with different extracts or PBS, and cultured. Cell viability and cytotoxicity were assessed at 24, 48, and 72 hours using CCK‐8(MedChemExpress LLC, Shanghai, China) and calcein/PI assays (Beyotime Biological Reagent Co. Ltd, Shanghai, China), analyzed with a gel analyzer (Universal Hood III, Burroughs, USA) and fluorescence microscope (Ti‐U, Nikon, Japan).

To test the biocompatibility in vivo of STZS_12_ and PSTZS_12_ sutures, animal study protocol was conducted ethically and approved by Jiangnan University's Animal Ethics Committee (JN. No20240630S0800915 [372]). SPF‐grade male Sprague‐Dawley rats were used, and 2 cm incisions were made on their dorsal area for subcutaneous implantation of STZS_12_ or PSTZS_12_ suture segments under aseptic conditions. Rats were euthanized at 3, 7, 14 and 28 days postoperatively, and the sutures with surrounding tissues were harvested and fixed in 4% paraformaldehyde (Beyotime Biological Reagent Co. Ltd, Shanghai, China). Specimens were divided into two groups for histological examination: one for HE staining after paraffin embedding, and the other for scanning electron microscopy after freeze‐drying. After 28 days, the rats were euthanized, and the hearts, livers, spleens, lungs, and kidneys were harvested from normal rats as well as those implanted with STZS_12_ and PSTZS_12_ sutures. These organs were fixed in paraformaldehyde and embedded in paraffin before being subjected to hematoxylin and eosin (H&E) staining to assess the organ toxicity of the sutures.

To verify the effect of sutures in promoting the migration of fibroblasts, we prepared extracts by combining sterilized samples with DMEM, ultrasonicated, incubated, and then filtered. A six‐well plate was prepared with L929 fibroblasts, cultured for 24 h, then scratched with a pipette tip [76]. After washing with PBS, DMEM and immersion solutions were added respectively. Reference images were taken at 0 hours, and the cultures were incubated for another 24 h. The scratch widths were monitored, photographed, and analyzed using Image J software.

To temporally resolve the perilesional accumulation of the active pharmaceutical ingredients, a standardized 2 cm×3 mm full‐thickness dorsal incision was created in adult rats and apposed with PSTZS_12_ sutures. On post‐operative days 1–4, contiguous 2 cm×1 cm skin biopsies centred on the wound margin were harvested, immersed in PBS (24 h, 37°C, constant agitation) to elute the analytes, and centrifugally clarified. Calibration curves for prilocaine (λ_max_ = 290 nm) and Zn^2+^(λ_max_ = 620 nm) were generated with certified standards; eluates were analysed in duplicate by UV–Vis spectrophotometry at the respective wavelengths to quantify optical density values proportional to drug concentration

### 4.5 In Vitro Antimicrobial Assay

Bacterial cultures of *Staphylococcus aureus* (ATCC 25923) and *Escherichia coli* (ATCC 25922) were provided by Jiangnan University Hospital. Sterilized 40 cm sutures of various types were immersed in 5 ml sterile PBS at 37°C with shaking. Over three days, 2 ml samples were collected every 24 hours and replaced with 2 ml fresh PBS.

*S. aureus* and *E. coli* were inoculated at 1 × 10^5^ CFU/ml onto LB agar. Sterilized swabs applied bacteria onto the medium 10 µl of the extracted solution was placed on 6 mm drug sensitivity paper disks daily. These disks were put on agar plates with bacteria and incubated at 37°C with 5% CO_2_ for 12 hours [77, 78, 79]. After incubation, inhibitory zones were documented using a Full‐featured Gel Imager. and measuring its diameter of range with Image J software.

To verify bacterial adhesion to sutures, 2 cm suture segments of various types were placed on agar plates with *Staphylococcus aureus* and *Escherichia coli*, then incubated at 37°C with 5% CO_2_ for a day. After incubation, the segments were fixed in 4% paraformaldehyde for 2 hours, rinsed thrice with deionized water, freeze‐dried, and analyzed using SEM to examine their topography and microbial interactions.

### 4.6 In Vivo Quadriceps Analgesia Experiment

To explore the effects of pain on the behavioral science of rats. Sprague‐Dawley rats were given an intraperitoneal injection of tribromoethanol at 25 mg/kg for anesthesia. Once anesthetized, the rats were positioned prone, and a 1 cm incision was made on the lateral side of the left thigh's quadriceps to expose the lateral femoral muscle after removing fat tissue.

A 3 mm deep and 8 mm long transverse incision was made in the midsection of the muscle using a scalpel. The muscle was sutured with STZS_12_ or PSTZS_12_, and the skin was closed with 5‐0 Mersilk. In the sham group, only the skin incision was closed. All surgeries were conducted under sterile conditions. Post‐surgery, rats' locomotor behavior, including walking speed, stride length, and standing time, was observed and recorded. Walking was captured via a camera in a transparent tube, and standing time was noted using an infrared positioner [80]. (Video S6 and S7)

In our study on glucose metabolism in the rat brain using a lateral femoral muscle incision model, we employed ^18^F‐FDG imaging. Rats fasted for 12 hours before intravenous injection of ^18^F‐FDG (a radioactive glucose analog) at 450 mCi via the tail vein. After a 1‐hour uptake period, 10‐min static PET brain scans were performed using a Super Nova micro‐PET/CT scanner (energy range: 350–650 keV; timing resolution: 2.56 ns). CT scans followed at 60 kVp, 500 µA, and 320 ms exposure time, with a 352 × 352 × 536 image matrix and 0.23 × 0.23 × 0.23 mm voxel size. Image reconstruction used a 3D OSEM/MAP algorithm (2 OSEM, 18 MAP iterations) with scatter and attenuation corrections, resulting in a 256 × 256 × 159 matrix and 0.39 × 0.39 × 0.80 mm voxel size [81].

^18^F‐FDG PET data were analyzed using PMOD software (v3.6). VOI analysis extracted data from 58 brain regions defined by a rat brain template and atlas, assigning SUVs to each region. Whole‐brain normalization was performed using the atlas. Regional SUVs were calculated by dividing each region's ^18^F‐FDG uptake by total brain uptake, enabling inter‐group comparisons [82, 83].

Regional SUVs were calculated by dividing the ^18^F‐FDG uptake in each region by the total brain uptake, as represented by formula (2).

(2) $$\mathrm{SUV}\;=\frac{Rb}{\frac{Rt}{W}}$$

Rb (Bq) denotes the radioactivity of ^18^F‐FDG uptake in brain region b, Rt (Bq) denotes the total brain radioactivity, and W (kg) denotes the rat's weight.

(3) $$Z\;=\frac{SUV_{experimental}-SUV_{normal}}{\delta }$$

where **δ** represents the discrete value of baseline SUVs.

A database with data from 12 untreated normal rats was created as a control. Differences in SUV were analyzed using Z scores (As shown in formula 3). Regions with |Z| > 3 were considered significantly different. Areas with Z > 3 were highlighted in red for increased uptake and Z < ‐3 in blue for decreased uptake, aiding in identifying significant metabolic changes in the brain.

### 4.7 Rat Dorsal Skin Incision Model and HE and Masson's Trichrome Staining, CD31/α‐SMA Staining

Sprague‐Dawley rats were anesthetized with tribromoethanol at a dose of 25 mg/kg and positioned prone. A 2 cm long and 2 mm deep incision was made on the dorsal skin and sutured with 5‐0 Mersilk, STZS_12_, and PSTZS_12_ sutures. Wound healing was observed on postoperative days 1, 3, 5, 7, 10, and 14, and skin incision tissues were collected on the corresponding days for histological examination. The tissues were fixed in 4% paraformaldehyde, dehydrated, and embedded in paraffin. Thin sections of 4 micrometers were cut and stained with hematoxylin and eosin (HE). To assess the suture's wound‐healing effects, skin tissues from days 7 and 14 were further subjected to HE and Masson's trichrome staining, as well as immunohistochemical staining for CD31 and α‐SMA, to evaluate the healing progress and the interactions between the suture materials and the skin.

### 4.8 Statistical Analysis

In this study, data were expressed as mean±standard deviation (SD) and analyzed using one‐way ANOVA to compare means across multiple groups. Significance levels were indicated by asterisks: * (p < 0.05), ** (p < 0.01), *** (p < 0.001), and **** (p < 0.0001), reflecting increasing levels of significance.

## Funding

The present study was funded by the Engineering Research Center of Wound Repair Technology, Ministry of Education (Grant No. K2050205‐01).

## Conflicts of Interest

The authors declare no conflict of interest.

## Supporting information




**Supporting File 1**: adhm71277‐sup‐0001‐VideoA.mp4.

**Supporting File 2**: adhm71277‐sup‐0002‐VideoB.mp4.

**Supporting File 3**: adhm71277‐sup‐0003‐Video2.mp4.

**Supporting File 4**: adhm71277‐sup‐0004‐Video3.mp4.

**Supporting File 5**: adhm71277‐sup‐0005‐Video4.mp4.

**Supporting File 6**: adhm71277‐sup‐0006‐Video5.mp4.

**Supporting File 7**: adhm71277‐sup‐0007‐Video6.mp4.

**Supporting File 8**: adhm71277‐sup‐0008‐Video7.mp4.

**Supporting File 9**: adhm71277‐sup‐0009‐SuppMat.docx.

## Data Availability

The data that support the findings of this study are available from the corresponding author upon reasonable request.
