# Supplementary material for: Mimetic Core‐Sheath Silk Sutures with Superior‐Strength for Pain Management, Anti‐Infection and Wound Healing
Source: Adv Healthc Mater. 2026 Jul 11;15(29):e00024. doi: 10.1002/adhm.202600024 (PMC13447971; doi:10.1002/adhm.202600024)
Supplement: Supplementary file 1 [file ADHM-15-0-s007.docx]

Supplementary Figure1

Supplementary Figure1 Mechanical properties of water-swollen sutures under high-humidity conditions.A. Stress-strain curves for 5-0 Mersilk^®^, STZS_12_, and PSTZS_12_.B. Stress statistics for 5-0 Mersilk^®^, STZS_12_, and PSTZS_12_.C. Strain statistics for 5-0 Mersilk^®^, STZS_12_, and PSTZS_12_.(All data presented as mean±SD, N=3)

Supplementary information 1

Specifically, the β-sheet structure of SerMA^[1]^ is identified by the amide I absorption peak at 1630 cm^-1^. In the case of raw SF, several key peaks are observed: the β-sheet structures of amide I, amide II, and amide III are represented by peaks^[2]^ at 1630 cm^-1^, 1510 cm^-1^, and 1230 cm^-1^, respectively. Additionally, the peak at 3280 cm^-1^ is attributed to the -OH vibration or stretching^[3]^. Meanwhile, the characteristic peaks^[4]^ of Prilocaine are also captured, revealing its unique spectral signature, include 3270 cm^-1^ for the N-H stretching vibration of the secondary amine, 3050 cm^-1^ for the N-H stretching vibration of the amide, 2938 cm^-1^ for the C-N stretching vibration of the amide, and 1680 cm^-1^ for the C=O stretching vibration of the amide. The C-H peaks on the benzene ring are identified at 1659 cm^-1^, 1590 cm^-1^, 1460 cm^-1^, 1120 cm^-1^, and the characteristic absorption peaks on the benzene ring (1,2-neighboring substituents) are at 750 cm^-1^.

The characteristic bands of PSTZS_12_, STZS_12_, TZS_12_, and TS_12_ are detailed in Figure 5D. Peaks at 1630 cm^-1^, 1510 cm^-1^, and 1230 cm^-1^ in TS_12_ represent the β-sheets of amide I, amide II, and amide III, respectively. In STZS_12_ and TZS_12_, the peak at 542 cm^-1^ is indicative of the Zn-O characteristic bond^[5]^. PSTZS_12_ exhibits characteristic peaks for silk protein, SerMA, Zn-O, and Prilocaine.

**Supplementary videos**

| Title | Video |
| --- | --- |
| Wet-spinning | Supplementary video 1A，Supplementary video 1B |
| Zinc ion loading | Supplementary video 2 |
| Mechanical testing of the suture | Supplementary video 3 |
| PET-CT 3D Modeling | Supplementary video 4 |
| Fiber Drawing | Supplementary video 5 |
| Tube Walking Test | Supplementary video 6 |
| Standing Test | Supplementary video 7 |

[1]	S. Ratanabunyong, J. Siriwaseree, P. Wanaragthai, S. Krobthong, Y. Yingchutrakul, B. Kuaprasert, K. Choowongkomon, P. Aramwit, *Sci Rep* **2024**, *14* (1), 2366.

[2]	L. Shu, P. Luo, Q. Chen, J. Liu, Y. Huang, C. Wu, X. Pan, Z. Huang, *Int J Pharm* **2024**, *664*, 124582.

[3]	X. Li, Y. Luo, F. Yang, G. Chu, L. Li, L. Diao, X. Jia, C. Yu, X. Wu, W. Zhong, M. Xing, G. Lyu, *Composites Part B: Engineering* **2023**, *260*.

[4]	B. V. Muniz, D. Baratelli, S. Di Carla, L. Serpe, C. B. da Silva, V. A. Guilherme, L. N. M. Ribeiro, C. M. S. Cereda, E. de Paula, M. C. Volpato, F. C. Groppo, L. F. Fraceto, M. Franz-Montan, *Sci Rep* **2018**, *8* (1), 17972.

[5]	Y. Xiao, Z. Yang, B. Guo, B. Wu, R. Liu, S. Zhang, P. Zhao, J. Ruan, X. Lu, K. Liu, D. Chen, *Advanced Functional Materials* **2023**, *34* (14).
